# Brain Hemodynamic Intermediate Phenotype Links Vitamin B_12_ to Cognitive Profile of Healthy and Mild Cognitive Impaired Subjects

**DOI:** 10.1155/2019/6874805

**Published:** 2019-06-02

**Authors:** Luca Cecchetti, Giada Lettieri, Giacomo Handjaras, Andrea Leo, Emiliano Ricciardi, Pietro Pietrini, Silvia Pellegrini, The Train the Brain Consortium

**Affiliations:** ^1^MoMiLab, IMT School for Advanced Studies Lucca, Lucca, Italy; ^2^Department of Clinical and Experimental Medicine, University of Pisa, Pisa, Italy; ^3^Institute of Clinical Physiology of the CNR, Via G. Moruzzi 1, 56100 Pisa, Italy

## Abstract

Vitamin B_12_, folate, and homocysteine are implicated in pivotal neurodegenerative mechanisms and partake in elders' mental decline. Findings on the association between vitamin-related biochemistry and cognitive abilities suggest that the structural and functional properties of the brain may represent an intermediate biomarker linking vitamin concentrations to cognition. Despite this, no previous study directly investigated whether vitamin B_12_, folate, and homocysteine levels are sufficient to explain individual neuropsychological profiles or, alternatively, whether the activity of brain regions modulated by these compounds better predicts cognition in elders. Here, we measured the relationship between vitamin blood concentrations, scores at seventeen neuropsychological tests, and brain activity of sixty-five elders spanning from normal to Mild Cognitive Impairment. We then evaluated whether task-related brain responses represent an intermediate phenotype, providing a better prediction of subjects' neuropsychological scores, as compared to the one obtained considering blood biochemistry only. We found that the hemodynamic activity of the right dorsal anterior cingulate cortex was positively associated (*p* value < 0.05 cluster corrected) with vitamin B_12_ concentrations, suggesting that elders with higher B_12_ levels had a more pronounced recruitment of this salience network region. Crucially, the activity of this area significantly predicted subjects' visual search and attention abilities (*p* value = 0.0023), whereas B_12_ levels *per se* failed to do so. Our results demonstrate that the relationship between blood biochemistry and elders' cognitive abilities is revealed when brain activity is included into the equation, thus highlighting the role of brain imaging as intermediate phenotype.

## 1. Introduction

Vitamin deficiencies due to dietary habits, drug interactions, or genetic factors are quite common in the elderly and may contribute to the worsening of cognitive decline [1, 2]. For instance, vitamin B_12_ and folate are involved in the metabolism of homocysteine (tHcy) [2, 3], and dysregulations in this system are related to mental decay in elders, being also involved in pivotal neurodegenerative mechanisms such as oxidative stress and the triggering of apoptosis [4]. The NHANES [5] and the Framingham Heart Study [6] demonstrated that high folate levels or folic acid supplementation might aggravate cognitive impairment associated with low vitamin B_12_ serum levels. Furthermore, high tHcy concentrations, together with folate and vitamin B_12_ deficiencies, are related to cerebrovascular pathologies [7] and accelerate brain ageing [8].

Despite the large amount of studies pointing to the same direction, other observations cast doubt on the reliability of the relationship between vitamin-related compounds and cognitive status [9]. For instance, a large cohort study on Alzheimer's disease and Mild Cognitive Impairment (MCI) patients did not find any correlation between cognitive functioning and vitamin B_12_, folate, or tHcy levels [10]. Similarly, in a double-blind randomized placebo-controlled trial, Dangour et al. did not find any positive effect of the supplementation of vitamin B_12_ on cognition [11]. Nonetheless, this trial included a limited number of subjects (*n* = 99) with no sign of cognitive decline, and the treatment was administered for a relatively brief period. Furthermore, a very recent investigation [12] showed no significant association between vitamin B_12_ levels and cognitive abilities in healthy elders. Overall, as there is a considerable heterogeneity in the cognitive and biochemical characteristics of samples between reports, further investigations are required to properly assess the relationship between blood biomarkers and cognition in elders [13, 14].

To clarify whether and how vitamin-related compounds affect the cognitive status of elders, it may be necessary to consider also information related to brain structure and function as measured by *in vivo* neuroimaging techniques. Indeed, brain properties may represent a compelling biomarker able to reveal how blood biochemistry contributes to cerebrovascular health and affects cognition. Accordingly, some studies provided indirect evidence of the interconnection between vitamin-related markers and behavior through the evaluation of brain structural properties: global and hippocampal atrophy are associated to high concentrations of tHcy [15, 16] and folate [15], while high B_12_ levels to decreased rates of total brain volume loss through the years [8, 17]. Of note, these structural alterations have been described not only in demented individuals but also in healthy elders [18, 19], indicating that blood biochemistry significantly influences brain morphology, regardless of the clinical diagnosis.

**Figure 1:**
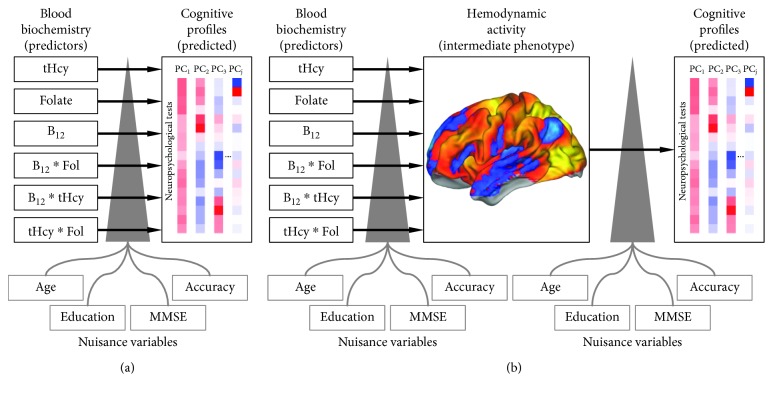
The two alternative hypotheses for the relationship between blood biochemistry, brain activity, and cognitive profiles. (a) Scheme depicting the potential role of B_12_, folate, tHcy, and their interactions as direct predictors of distinct cognitive profiles. These profiles are represented by principal components (PC) derived from subjects' scores at seventeen neuropsychological tests. Age, years of education, Mini Mental State Examination scores and fMRI task accuracy are included in the model as nuisance variables. (b) Alternatively, brain hemodynamic activity can act as an intermediate phenotype in linking blood biochemistry to subjects' cognitive profiles. First, brain regions modulated by B_12_, folate, tHcy, and their interactions are identified and hemodynamic activity is subsequently used as a predictor of cognitive profiles. The same nuisance variables are included in the model at both steps of analysis. tHcy: homocysteine; Fol: folate; MMSE: Mini Mental State Examination.

However, in sharp contrast with the abundance of evidence linking biochemical markers to brain morphological alterations, less is known about the relationship with brain functioning [20, 21]. Most importantly, to the best of our knowledge, no previous study directly investigated whether levels of vitamin-related compounds are sufficient to explain cognition (Figure 1(a)) or, alternatively, brain hemodynamic properties act as an intermediate phenotype providing a significant prediction of subjects' cognitive profiles (Figure 1(b)). Thus, to fill this gap in the literature, we measured the correlation between vitamin B_12_, folate, and tHcy blood levels and brain activity during a visuospatial attention task [22] in a group of sixty-five nondemented elders, with normal cognition or MCI diagnosis. We then ascertained whether brain regions significantly modulated by concentrations of vitamin-related compounds predicted subjects' cognitive profiles, as measured through a comprehensive neuropsychological assessment.

## 2. Methods

### 2.1. Subjects

From the initial cohort of a longitudinal project [22] (the Train the Brain study; ClinicalTrials.gov Identifier: NCT01725178), we selected subjects who completed the neuropsychological, biochemical, and MRI assessment (see Supplementary Figure 1 for a recruitment flowchart). Seventy-four elders were then included in the current study (35 males; mean age ± standard deviation: 74 ± 5 years; education: 10 ± 5 years; Mini Mental State Examination (MMSE) score: 26.1 ± 2.1). Neuropsychological and neurological evaluations assessed subjects' cognitive and physical state and excluded a diagnosis of moderate or severe dementia (Clinical Dementia Rating > 1) and depression (Geriatric Depression Scale ≥ 9), as well as other psychiatric, neurological, or medical conditions.

In accordance with the European Consortium on Alzheimer's Disease Working Group on MCI criteria [23], participants' cognitive state ranged from normal (*n* = 18) to MCI (*n* = 56).

Subjects gave their written informed consent to take part in the study and had the right to withdraw at any time. Protocol and procedures were approved by the local Ethical Committee for Clinical Experimentation and the study was conducted in accordance with the Declaration of Helsinki.

Prior to the analyses, we discarded nine subjects due to the following reasons: (i) their B_12_, folate, and tHcy values were higher or lower than 3 standard deviations from the group average (recursive outlier detection procedure; *n* = 5) and (ii) they had excessive head movement during fMRI acquisition (*n* = 4; see MRI Data Analysis). Thus, the data of 65 elders have been used to test the relationship between blood biochemistry, brain activity, and cognitive profiles: 30 males; mean age ± standard deviation: 74 ± 5 years; education: 10 ± 5 years; and MMSE score: 25.9 ± 2.0. There were 15 healthy elders (HE) and 27 elders with amnestic single-domain MCI, 16 with amnestic multiple domain MCI, five with nonamnestic single-domain MCI, and two with nonamnestic multiple domain MCI.

### 2.2. Data Acquisition

#### 2.2.1. Neuropsychological Assessment

To characterize the cognitive state of our sample, expert neuropsychologists administered a comprehensive battery of 18 tests, investigating multiple cognitive domains. A detailed description is provided in Supplementary Materials. Sample characteristics and test results are reported in Supplementary Table 1.

#### 2.2.2. Clinical Biochemistry Evaluation

Subjects underwent a fasting blood sampling procedure within one week prior to MRI acquisition. Blood analyses were performed by the Clinical Biochemistry Laboratory of the Azienda Ospedaliera Universitaria Pisana (Pisa, Italy) which also included routine blood tests. B_12_ and folate serum levels were evaluated by an electrochemiluminescence immunoassay (ECLIA) (Roche Diagnostics, Basilea, Switzerland) on a cobas immunoanalyzer (Roche Diagnostics, Basilea, Switzerland), whereas tHcy was evaluated in plasma by the automated latex enhanced immunoassay HemosIL (Werfen, Barcelona, Spain) on an ACL TOP 500 instrument (Werfen, Barcelona, Spain).

#### 2.2.3. Magnetic Resonance Imaging

MRI data were acquired on a GE HDxt 1.5 T Signa (General Electric Healthcare) system, equipped with an 8-channel phased-array head coil. For each subject, an exhaustive MR session was performed, including a clinical protocol (i.e., T2w FSE, FLAIR, and T2∗GRE) reviewed by an expert neuroradiologist that ensured the absence of pathological conditions. A T1-weighted 3D fast spoiled gradient recall sequence (TR/TE = 12.650/5.300 ms, prep time = 700 ms, NEX = 1, FOV = 256 mm, acquisition matrix = 256 × 256, isotropic voxel = 1 × 1 × 1 mm, and approximately 10 minutes of scan time) provided high-resolution structural images of the brain, whereas a gradient recall echo-echo planar imaging sequence (TR/TE = 2500/50 ms; FA = 90°; FOV = 192 mm; acquisition matrix = 64 × 64; isotropic voxel = 3 × 3 × 3 mm; 33 interleaved axial slices; partial brain coverage: ~10 cm; three runs; 60 + 4 dummy volumes each; and 8-minute overall scan time) measured hemodynamic responses elicited by a visuospatial attention task [22]. During the MR session, participants wore MR-compatible goggles (VisuaStim, Resonance Technology Inc.; 30 × 22.5 deg visual field size) that allowed the projection of visual stimuli. Two buttons (held in the left and right hands) were also provided and used to track the subject' responses during task execution.

#### 2.2.4. Stimuli and Experimental Paradigm

The experimental apparatus (i.e., goggles and response pads) was connected to a workstation running MATLAB Release 2010b 64 bit (The MathWorks Inc., Natick, MA, USA). The visuospatial attention task was implemented in the Psychtoolbox v3.0.947 [24] and administered during the fMRI acquisition. In brief, subjects were asked to covertly track four stimuli (i.e., two red and two blue dots) while gazing on a central fixation point. The four dots were moving randomly at a constant speed of 6 deg/s. Two near-peripheral (i.e., 7.5 deg of eccentricity) colored targets were located to the right (i.e., red target) and to the left (i.e., blue target) of the fixation point. Participants had to press the correct response button whenever one stimulus hit the target of the same color and their performance was recorded in real-time (see Supplementary Figure 2). Overall, subjects had to accomplish nine repetitions of the task across three runs. Hence, each run comprised three blocks of tasks, lasting 30 seconds each, alternated with four resting intervals (15 seconds each). During rest periods, only the two static targets were presented and subjects were instructed to gaze the central fixation point, without focusing on specific thoughts. To familiarize subjects with task procedure, a brief training session was provided outside the scanner room, which ensured all participants to achieve at least 66% accuracy. Biochemical, neuropsychological, and MRI data are available upon request.

### 2.3. Data Analysis

#### 2.3.1. Neuropsychological Assessment

Scores at neuropsychological tests were adjusted for age and education and transformed into *Z*-scores (excluding the MMSE). Pearson's coefficient estimated the correlation among them, and the level of significance was corrected for multiple comparisons through the False Discovery Rate procedure [25] (*p* value_FDR_ < 0.05). Principal component analysis was implemented in MATLAB (release 2010b 64 bit) and led to 17 linearly uncorrelated dimensions (i.e., cognitive profiles), each representing an aggregate measure of subjects' performance at several tests. This approach was employed to solve the high collinearity in the neuropsychological evaluation, since each test engages multiple cognitive systems [26]. Given that PC scores are, by definition, uncorrelated to each other, they were used to assess the relationship between blood biochemistry, task-evoked brain activity, and cognitive profiles using independent tests.

#### 2.3.2. Clinical Biochemistry Evaluation

In our final sample (*n* = 65), the average level (±standard deviation) of vitamin B_12_ was 400.6 ± 145.7 pg/mL (range: 124-779 pg/mL), whereas for folate and tHcy the average values were 6.7 ± 2.2 ng/mL (range: 3.2-12.0 ng/mL) and 10.2 ± 3.4 *μ*mol/L (range: 4.0-18.8 *μ*mol/L), respectively. B_12_, folate, and tHcy values were transformed into *Z*-scores and interaction terms were estimated: tHcy∗folate, tHcy∗B_12_, and folate∗B_12_. Negative scores for the interaction terms reflected a valuable imbalance between blood levels of vitamin-related compounds, whereas positive values indicated that both biochemical markers were either above or below average. As previous evidence revealed the existence of a relationship between these compounds and age [18], MMSE scores [27], and clinical diagnosis [28], we performed a comprehensive set of supporting analyses, detailed in Supplementary Materials.

Moreover, we evaluated whether vitamin-related compounds would significantly predict subjects' cognition, as measured by PCs (Figure 1(a)). Seventeen independent general linear models (GLMs) verified this relationship: blood marker concentrations and their interactions were treated as variables of interest, while age, years of education, MMSE scores, and fMRI task accuracy were treated as nuisance variables. The assessment of statistical significance was performed using a nonparametric permutation approach, as detailed below (see MRI Data Analysis). Results were corrected for multiple comparisons using the Bonferroni method (Bonferroni corrected *p* value < 0.05; *p*_crit_ = 0.0029).

#### 2.3.3. fMRI Task Performance

In the whole sample, ranging from healthy to mild cognitive impaired subjects, we tested the relationship between fMRI task performance and age, MMSE scores, clinical diagnosis, and blood biochemistry (see Supplementary Materials). Furthermore, we used 17 independent GLMs to measure the relationship between fMRI task performance and PC scores, while adjusting the results for age, years of education, and MMSE (Bonferroni corrected *p* value < 0.05; *p*_crit_ = 0.0029).

#### 2.3.4. MRI Data Analysis

MRI data were extensively preprocessed to rule out potential confounds related to physiological, as well as acquisition-related artifacts, and analyzed using ANTs [29], AFNI v.17.2.00 [30], and FSL v.5.0.9 [31]. For a comprehensive description of single-subject processing steps, please refer to Supplementary Materials. Group-level correlation between brain activity and blood biochemistry was evaluated through a mixed-effect model (3dMEMA), which considered within- and between-subject variability. B_12_, folate, tHcy, and the interaction terms represented the regressors of interest for the group-level analysis, while *Z*-transformed age, education, MMSE, and task accuracy at the visuospatial attention task were included as confounds. Significance of the group-level correlation was corrected for multiple comparisons through AFNI's cluster-based method (3dClustSim; two-tailed *p* value < 0.005 for the cluster-forming threshold and *p* value < 0.05 for cluster significance), considering the average spatial autocorrelation of the data across subjects.

Lastly, we assessed whether cerebrovascular activity acts as an intermediate phenotype, indirectly linking blood biochemistry to cognition in nondemented elders. To this purpose, the activity of brain regions showing significant group-level correlation with vitamin-related compounds was used as a predictor of cognitive profiles. Thus, for each of the identified regions, we performed 17 independent GLMs using subjects' hemodynamic activity as the explanatory variable. Age, years of education, and MMSE scores as well as fMRI task accuracy were included as nuisance regressors and each PC score was included as the response variable. To robustly estimate the statistical significance of each test, we adopted a permutation approach in which both dependent and independent matrices of the GLMs were rearranged (100,000 permutations) by shuffling values within each column (i.e., permuting subject ID), separately. This procedure generated a null distribution of regression coefficients, against which the actual association was tested. The resulting *p* values were corrected for multiple comparisons through the Bonferroni method (Bonferroni corrected *p* value < 0.05; *p*_crit_ = 0.0029).

## 3. Results

### 3.1. Neuropsychological Assessment

**Figure 2:**
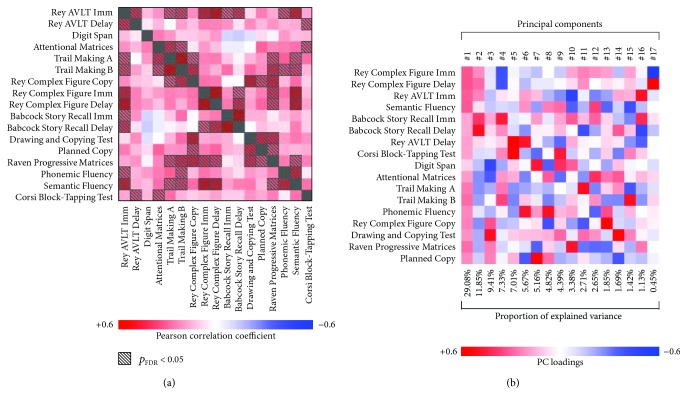
Neuropsychological assessment results. (a) Correlations among all the seventeen neuropsychological tests as estimated by Pearson's coefficient. Marked cells refer to significant correlations corrected for multiple comparisons through the False Discovery Rate procedure. (b) Matrix describing the results of the principal component analysis and showing seventeen uncorrelated cognitive profiles, tests loadings, and the proportion of variance explained by each component. As a matter of fact, the first principal component (explained variance: 29.1%) highlighted subjects' global cognitive performance, since all the neuropsychological tests jointly contributed to it and given its high correlation with MMSE scores (*r* = 0.522, *p* value = 8.2∗10^−6^). AVLT: Auditory Verbal Learning Test; Imm: immediate recall; Delay: delayed recall.

As depicted in Figure 2(a) and detailed in Supplementary Materials, we observed several significant correlations among scores at different neuropsychological tests. Principal components (i.e., cognitive profiles) were computed and interpreted according to the loadings of the seventeen cognitive tests (Figure 2(b)).

### 3.2. Clinical Biochemistry Evaluation

Results for the relationship between blood biochemistry and subjects' cognitive performance as measured by the PCs are shown in Table 1 and Supplementary Figures 4(A) and 5(A). We found a negative association between working memory abilities versus lexical access speed (Figure 2(b); 12th PC) and both folate and B_12_∗folate levels, as well as a positive association between tHcy∗folate interaction and attention performance versus verbal fluency scores (Figure 2(b); 15th PC). Besides these, neither additional biochemical markers, nor other interactions, significantly predicted any cognitive profile (Supplementary Table 2).

### 3.3. fMRI Task Performance

Average task performance ± standard deviation across individuals was 80.3% ± 8%. None of the linear associations between fMRI task accuracy and PCs reached statistical significance (see Supplementary Table 7). Results from other supporting analyses are detailed in Supplementary Materials.

### 3.4. MRI Data

**Figure 3:**
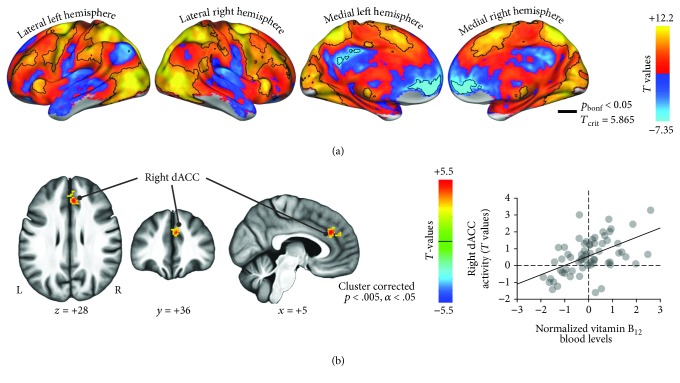
Brain activity results. (a) fMRI task-evoked activity across subjects entails medial and lateral occipital regions (i.e., primary and motion-sensitive visual cortex), the dorsal parietal attention network (intraparietal sulcus and superior parietal lobule), frontal areas involved in motor control and focusing (ventral and dorsal premotor, as well as the supplementary motor), and nodes of the salience network (e.g., anterior insula). Regions enclosed in black survived the Bonferroni correction for multiple comparisons (*p* value < 0.05). (b) Results for the relationship between biochemical markers and task-evoked brain activity. Hemodynamic activity of the right dorsal anterior cingulate cortex (dACC), located within the task-positive network, was found to be significantly associated with B_12_ serum concentrations (two-tailed *p* value < 0.005 for the cluster-forming threshold and *α* < 0.05 for cluster significance).

Overall, during the execution of the visuospatial attention task, participants recruited a widespread bilateral network (Figure 3(a)).

**Figure 4:**
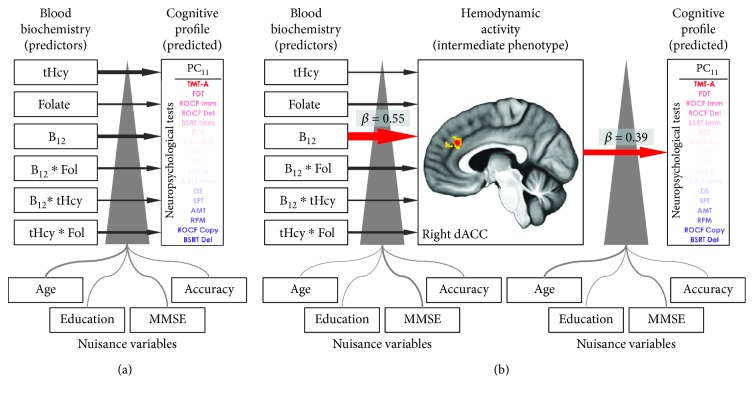
Resulting model for the relationship between blood biochemistry, right dACC activity, and cognitive profiles. (a) Scheme depicting the role of B_12_, folate, tHcy, and their interactions, as direct predictors of the cognitive profile expressed by the 11th component (PC). No significant associations were found between biochemical markers and cognitive status. Loadings of each neuropsychological test for the 11th component are color coded: the more intense the red is, the more positive is the loading of a test; vice versa, the more intense the blue is, the more negative the loading. Age, years of education, Mini Mental State Examination scores, and fMRI task accuracy are included in the model as nuisance variables. (b) Activity of the right dACC significantly acts as an intermediate phenotype in linking B_12_ serum levels to subjects' spatial attention and search abilities. Indeed, the right dACC hemodynamic activity correlated with B_12_ serum levels (*β* = 0.55) and predicted PC_11_ (*β* = 0.39). Please note that B_12_ levels per se were not able to predict the same cognitive profile. In both panels, arrow thickness represents the strength of the association between variables. Significant relationships are marked with colored arrows (positive relationships red, negative blue). The same nuisance variables are included in the model at both steps of analysis. RPM: Raven's Progressive Matrices; AVLT: Rey Auditory Verbal Learning Test; BSRT: Babcock Story Recall Test; DS: Forward Digit Span; AMT: Attentional Matrices Test; TMT: Trail Making Test; ROCF: Rey-Osterrieth Complex Figure; CBTT: Corsi Block-Tapping Test; FDT: Freehand Drawing Test; PCT: Planned Copy Test; PFT: Phonemic Fluency Test; SFT: Semantic Fluency Test; Imm: immediate recall; Del: delayed recall; tHcy: homocysteine; Fol: folate; MMSE: Mini Mental State Examination.

Interestingly, results for the relationship between vitamin-related compounds and brain activity highlighted a significant ROI within the task-positive network: hemodynamic activity of the right dorsal anterior cingulate cortex (dACC; center of gravity: *x* = +5, *y* = +36, *z* = +28; peak of association: *x* = +6, *y* = +34, *z* = +28) was positively associated with B_12_ serum concentrations (*β* = 0.551; Figures 3(b) and 4(b)). The average group-level signal of this area was positive during task blocks (+1.36%), suggesting that participants with higher B_12_ levels had a more pronounced recruitment of this salience network region [32].

**Figure 5:**
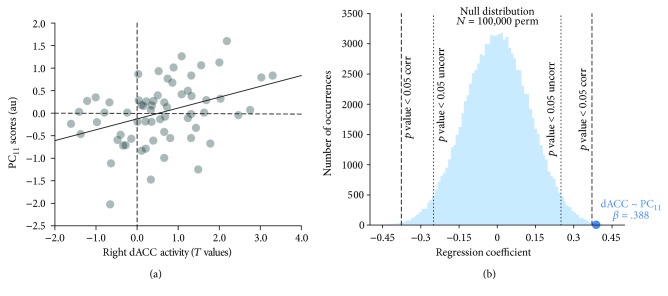
Predictive power of brain activity on cognitive profile. (a) Scatter plot depicting the relationship between hemodynamic activity of the right dorsal anterior cingulate cortex (dACC) and the cognitive profile expressed by the 11th component (PC), related to subjects' spatial attention and search abilities (i.e., scores at the Trail Making Test A versus delayed recall of the Babcock story). (b) Null distribution of regression coefficients against which the relationship between right dACC activity and cognitive profile expressed by the 11th component was tested. The blue dot indicates the actual relationship between the right dACC and the 11th PC; dotted lines represent *p* value < 0.05 uncorrected threshold, while dashed lines represent *p* value < 0.05 Bonferroni corrected.

Most importantly, dACC activity significantly predicted (*β* = 0.388, raw *p* value = 0.0023; Bonferroni corrected *p* value = 0.039; Figures 5(a) and 5(b); Figure 4(b); Table 2; Supplementary Table 3) the cognitive profile related to subjects' spatial attention and search abilities versus verbal memory performance (11th PC: Trail Making Test A loading = 0.512; Babcock delayed story recall loading = −0.411; Figure 2(b)). In addition, by considering B_12_ levels only it was not possible to explain subjects' performances for any of the cognitive profiles (see Supplementary Table 2 and Figure 4(a)). Of note, this result was not affected by the exclusion of healthy elders from the analysis: when considering MCI patients only (*n* = 50), the association between dACC activity and the 11th PC was still significant (*β* = 0.433, raw *p* value = 0.0027). Taken together, these results highlighted the role of task-positive brain activity as an intermediate phenotype, linking blood biochemistry to cognition throughout the normal-to-pathology continuum. Moreover, other regions of the task-negative network, modulated by vitamin-related compounds (Supplementary Figure 3), did not add a relevant contribution to the prediction of cognitive profiles, which can be solved by simply considering blood biochemistry (see Supplementary Materials).

## 4. Discussion

In the present study, we used magnetic resonance imaging, blood biochemistry, and neuropsychological assessment to investigate whether functional characteristics of the brain, mediated by B_12_, folate, and tHcy concentrations, represent an intermediate phenotype able to significantly predict single-subject cognitive profiles. Our results demonstrated that, in sixty-five nondemented elders performing a visuospatial attention task, B_12_ levels were positively associated to hemodynamic responses in the right dACC. Crucially, the activity of this brain region, but not B_12_ levels *per se*, predicted participants' spatial attention and visual search abilities as measured by an independent neuropsychological evaluation (Figure 4). This finding unveiled how B_12_ contributes to cerebrovascular health, indirectly affecting cognition and mental efficiency in healthy elders and in prodromal stages of dementia.

Some authors suggested that in old age B_12_ serum levels together with folate concentrations have a fundamental role in genomic and nongenomic methylation, and their deficiencies trigger homocysteine-mediated neurotoxicity [33]. In addition, the effects of this vitamin on cognition have been demonstrated throughout the normal-to-pathology continuum [1, 6].

This relationship has been further corroborated by reports linking B_12_ concentrations to brain structural properties, mainly focusing on global parenchymal atrophy and white-matter lesions. Vogiatzoglou et al. showed that healthy elders with lower B_12_ levels had higher global atrophy scores and increased rate of brain volume loss [34]. Studies have reported a protective effect of high blood concentrations [8] and dietary intake [19] of B_12_ on the brain atrophy rate in healthy elders, as well as in MCI patients [2]. Similarly, the severity of white-matter lesions is negatively related to vitamin concentrations [35], as testified also by a recent diffusion tensor imaging study [36].

Despite the abundance of structural MRI findings, less is known about how B_12_ affects brain functioning. To our knowledge, only two studies investigated this issue: one showed that regional homogeneity of resting state hemodynamic activity is reduced in patients suffering B_12_ deficiencies, especially within the default mode and the cinguloopercular and frontoparietal networks [20]. The other one demonstrates that the same patients exhibited reduced occipitoparietal and increased frontal cerebral blood flow values [21].

In the current report, we found that B_12_ serum concentrations are positively associated to the right dACC hemodynamic activity during a visuospatial attention task that engages attention-, action-planning-, and motor-control-related brain regions [22]. The dACC has rich connectivity with the anterior insular (AI) cortex through the uncinate fasciculus and many efferent motor projections [37]. Interestingly, together with AI, dACC is considered a crucial node of the salience network, which is part of the task-positive network [38] and sustains the detection of cognitively relevant events [32]. Indeed, the Neurosynth (http://old.neurosynth.org; [39]) association terms related to the peak coordinates of the right dACC were “salience” (posterior probability=0.74; *z* − score = 5.17) and “salience network” (posterior probability=0.82; *z* − score = 5.05). The salience network is thought to activate the executive network and to deactivate the default mode network during the execution of tasks oriented toward the environment [40–42]. Particularly, within the salience network, dACC is devoted to response selection [43] and conflict monitoring [44], two crucial activities for the proper execution of our visuospatial task. A previous study unveiled the relationship between dACC activity during a response inhibition task and clinical diagnosis: compared to Alzheimer's disease patients, MCI demonstrated a compensatory mechanism, leading to an increase in the hemodynamic activity of this region [45]. Evidence from resting state connectivity further highlighted the association between salience network integrity and overall cognitive state [46].

In our study, we aimed to overcome the dichotomy imposed by the clinical evidence and to ascertain whether the task-related brain activity mediates the effects of blood biochemistry on individual neuropsychological profiles, regardless of the diagnosis. It is worth mentioning that it was not possible to predict subjects' performance at neuropsychological tests by simply considering B_12_ serum concentrations (Figure 4(a)). However, by including brain activity in the equation, we were able to significantly estimate elders' cognition: individuals with higher spatial attention and visual search abilities (and lower verbal memory performances) have a more substantial recruitment of dACC, whose activity is positively modulated by B_12_ (Figure 4(b)). Interestingly, in our data only the right dACC acted as an intermediate phenotype connecting B_12_ concentrations to the single-subject cognitive state in healthy elders and in prodromal stages of dementia. The right lateralization of this finding is not surprising, as our task requires the engagement of visuospatial abilities, and the processing of the spatial characteristics of stimuli mainly relies on right hemisphere computations [47]. This general observation is supported by lesion studies and neuropsychological literature (see [48]), as well as by in vivo neuroimaging investigations [49, 50]. Other studies also demonstrated that this right lateralization holds for error processing and conflict monitoring [51], as well as for visuospatial processing [52] in dACC.

In addition, the inclusion of B_12_ as a confound in the correlation analysis between the right dACC and the cognitive profile did not alter the significance of their association (*β* = 0.392, raw *p* value = 0.0022; Bonferroni corrected *p* value = 0.037; Supplementary Table 5). This evidence clearly indicates that distinct mechanisms related to serum B_12_ concentrations and visual search abilities jointly influence dACC activity.

From a more general perspective, our results suggest that only task-positive brain activity significantly contributes to solve the indirect relationship between blood biochemistry and cognitive profiles, while the hemodynamic activity of task-negative regions may not (see also Supplementary Materials). Indeed, further studies including different fMRI paradigms (e.g., verbal naming and lexical decision) and blood tests are required to better characterize whether only the task-positive activity represents a proper brain marker, linking biochemistry to cognitive profiles. We trust that the adoption of other tasks (e.g., memory encoding) may unveil relationships between clinical biochemistry, the activity of specific brain regions (e.g., the hippocampus), and subjects' neuropsychological profiles (e.g., scores at verbal memory tests versus performance at executive functions tests).

Furthermore, it is relevant to note that subjects' accuracy at the visuospatial attention task recorded during fMRI acquisition did not correlate with any of the cognitive profiles, or with any of the vitamin-related compounds except for the B_12_∗tHcy interaction (*β* = 0.335, *p* value = 0.006; Supplementary Table 7). Hence, the role of dACC as a B_12_ intermediate phenotype does not simply depend on a direct association between subjects' abilities at two different tasks (i.e., the one designed for the fMRI experiment and Trail Making Test A) investigating the same cognitive domain (i.e., visuospatial abilities).

In summary, our study is the first to prove that modelling brain activity as an intermediate factor between biochemical markers and cognition unveils their indirect relationship. This evidence suggests that the endophenotypic approach, successfully adopted to explore associations between genes, brain, and complex behaviors [53], can be extended to biochemical factors, a possibility hitherto neglected.

## Figures and Tables

**Table 1:** Predictive power of biochemical markers on cognitive profiles.

	PC_1_		PC_7_		PC_12_		PC_15_	
	*β*	*p*	*β*	*p*	*β*	*p*	*β*	*p*
tHcy	.011	.9322	-.068	.6180	-.138	.3098	-.076	.5790
Folate	-.080	.5563	.021	.8841	**-.409**	**.0021**	.154	.2616
B_12_	.222	.1034	.171	.2071	-.147	.2746	.007	.9567
B_12_∗folate	.189	.1629	-.117	.3926	**-.480**	**.0002**	.012	.9254
B_12_∗tHcy	.037	.7844	.066	.6272	-.139	.3076	-.081	.5522
tHcy∗folate	.149	.2689	.007	.9604	-.253	.0618	**.428**	**.0011**
Age	-.154	.2596	**.431**	**.0009**	-.175	.2004	.038	.7802
Education	.101	.4625	*.346*	*.0097*	.233	.0840	.031	.8185
MMSE scores	**.553**	**.0000**	-.042	.7674	-.042	.7621	*.325*	*.0164*
fMRI task accuracy	.037	.7912	-.029	.8342	.126	.3566	-.060	.6629

Model: PC_j_=*α*+*β* tHcy+*β* folate+*β* B_12_+*β* B_12_∗folate+*β* B_12_∗tHcy+*β* tHcy∗folate+*β* age+*β* education+*β* MMSE+*β* fMRI task accuracy; italicized values represent *p* < 0.05 uncorrected; bold values represent *p* < 0.05 Bonferroni corrected; tHcy=homocysteine; MMSE=Mini Mental State Examination; PC=principal component.

**Table 2:** Predictive power of right dACC activity on cognitive profiles.

	PC_1_		PC_7_		PC_11_	
	*β*	*p*	*β*	*p*	*β*	*p*
Right dACC	.188	.1467	.210	.1049	**.388**	**.0023**
Age	-.233	.0713	**.446**	**.0003**	.142	.2737
Education	.104	.4276	*.356*	*.0047*	-.023	.8642
MMSE scores	**.565**	**.0000**	-.055	.6728	.113	.3870
fMRI task accuracy	-.001	.9918	-.045	.7291	.007	.9592

Model: PC_j_=*α*+*β* right dACC+*β* age+*β* education+*β* MMSE+*β* fMRI task accuracy; underlined values represent *p* < 0.05 uncorrected; bold values represent *p* < 0.05 Bonferroni corrected; dACC=dorsal anterior cingulate cortex; MMSE=Mini Mental State Examination; PC=principal component.

## Data Availability

The biochemical, neuropsychological, and MRI data used to support the findings of this study are available from the corresponding author upon request.

## Supplementary Materials

Supplementary 1 Supplementary Methods: a paragraph detailing the neuropsychological assessment, clinical biochemistry evaluation, and MRI data analysis. Supplementary Results: all the results that are not included in the main manuscript. Appendix: list of the members and affiliations of the Train the Brain Consortium. Supplementary Figure 1: the flowchart for the selection of participants. Supplementary Figure 2: the stimuli employed in the fMRI experiment. Supplementary Figure 3: additional results for the correlation between brain activity and blood biochemistry markers. Supplementary Figure 4: resulting model for the relationship between blood biochemistry, right vmPFC activity, and cognitive profiles. Supplementary Figure 5: resulting model for the relationship between blood biochemistry, bilateral vmPFC activity, and cognitive profiles. Supplementary References. Supplementary Table 1: demographic and neuropsychological data. Supplementary Table 2: predictive power of biochemical markers on cognitive profiles. Supplementary Table 3: predictive power of right dACC activity on cognitive profiles. Supplementary Table 4: predictive power of vmPFC activity on cognitive profiles. Supplementary Table 5: predictive power of right dACC activity on cognitive profiles, controlling for vitamin B_12_. Supplementary Table 6: predictive power of vmPFC activity on cognitive profiles, controlling for blood biochemistry. Supplementary Table 7: predictive power of fMRI task accuracy on cognitive profiles.

Supplementary 2 Complete list of the members and affiliations of the Train the Brain Consortium.

## References

[1] Moore E. M., Ames D., Mander A. G. (2014). Among vitamin B_12_ deficient older people, high folate levels are associated with worse cognitive function: combined data from three cohorts. *Journal of Alzheimer's Disease*.

[2] Smith A. D., Refsum H. (2016). Homocysteine, B vitamins, and cognitive impairment. *Annual Review of Nutrition*.

[3] Stabler S. P. (2013). Vitamin B_12_ deficiency. *New England Journal of Medicine*.

[4] Sachdev P. S. (2005). Homocysteine and brain atrophy. *Progress in Neuro-Psychopharmacology and Biological Psychiatry*.

[5] Yetley E. A., Pfeiffer C. M., Phinney K. W. (2011). Biomarkers of vitamin B-12 status in NHANES: a roundtable summary. *The American Journal of Clinical Nutrition*.

[6] Morris M. S., Selhub J., Jacques P. F. (2012). Vitamin B-12 and folate status in relation to decline in scores on the mini-mental state examination in the Framingham heart study. *Journal of the American Geriatrics Society*.

[7] Jeon S. B., Kang D. W., Kim J. S., Kwon S. U. (2014). Homocysteine, small-vessel disease, and atherosclerosis: an MRI study of 825 stroke patients. *Neurology*.

[8] Hooshmand B., Mangialasche F., Kalpouzos G. (2016). Association of vitamin B_12_, folate, and sulfur amino acids with brain magnetic resonance imaging measures in older adults: a longitudinal population-based study. *JAMA Psychiatry*.

[9] Ellinson M., Thomas J., Patterson A. (2004). A critical evaluation of the relationship between serum vitamin B_12_, folate and total homocysteine with cognitive impairment in the elderly. *Journal of Human Nutrition and Dietetics*.

[10] Arioğul S., Cankurtaran M., Dağli N., Khalil M., Yavuz B. (2005). Vitamin B_12_, folate, homocysteine and dementia: are they really related?. *Archives of Gerontology and Geriatrics*.

[11] Dangour A. D., Allen E., Clarke R. (2015). Effects of vitamin B-12 supplementation on neurologic and cognitive function in older people: a randomized controlled trial. *The American Journal of Clinical Nutrition*.

[12] Hughes C., Ward M., Tracey F. (2017). B-vitamin intake and biomarker status in relation to cognitive decline in healthy older adults in a 4-year follow-up study. *Nutrients*.

[13] Miles L. M., Mills K., Clarke R., Dangour A. D. (2015). Is there an association of vitamin B_12_ status with neurological function in older people? A systematic review. *British Journal of Nutrition*.

[14] Porter K., Hoey L., Hughes C., Ward M., McNulty H. (2016). Causes, consequences and public health implications of low B-vitamin status in ageing. *Nutrients*.

[15] Ford A. H., Garrido G. J., Beer C. (2012). Homocysteine, grey matter and cognitive function in adults with cardiovascular disease. *PLoS One*.

[16] Gallucci M., Zanardo A., Bendini M., di Paola F., Boldrini P., Grossi E. (2014). Serum folate, homocysteine, brain atrophy, and Auto-CM system: the Treviso dementia (TREDEM) study. *Journal of Alzheimer's Disease*.

[17] Jernerén F., Elshorbagy A. K., Oulhaj A., Smith S. M., Refsum H., Smith A. D. (2015). Brain atrophy in cognitively impaired elderly: the importance of long-chain *ω*-3 fatty acids and B vitamin status in a randomized controlled trial. *The American Journal of Clinical Nutrition*.

[18] den Heijer T., Vermeer S. E., Clarke R. (2003). Homocysteine and brain atrophy on MRI of non-demented elderly. *Brain*.

[19] Erickson K. I., Suever B. L., Prakash R. S., Colcombe S. J., McAuley E., Kramer A. F. (2008). Greater intake of vitamins B_6_ and B_12_ spares gray matter in healthy elderly: a voxel-based morphometry study. *Brain Research*.

[20] Gupta L., Gupta R. K., Gupta P. K., Malhotra H. S., Saha I., Garg R. K. (2016). Assessment of brain cognitive functions in patients with vitamin B_12_ deficiency using resting state functional MRI: a longitudinal study. *Magnetic Resonance Imaging*.

[21] Roy B., Trivedi R., Garg R. K., Gupta P. K., Tyagi R., Gupta R. K. (2015). Assessment of functional and structural damage in brain parenchyma in patients with vitamin B_12_ deficiency: a longitudinal perfusion and diffusion tensor imaging study. *Magnetic Resonance Imaging*.

[22] Train the Brain Consortium (2017). Randomized trial on the effects of a combined physical/cognitive training in aged MCI subjects: the Train the Brain study. *Scientific Reports*.

[23] Portet F., Ousset P. J., Visser P. J. (2006). Mild cognitive impairment (MCI) in medical practice: a critical review of the concept and new diagnostic procedure. Report of the MCI Working Group of the European Consortium on Alzheimer’s Disease. *Journal of Neurology, Neurosurgery & Psychiatry*.

[24] Kleiner M., Brainard D., Pelli D., Ingling A., Murray R., Broussard C. (2007). What’s new in psych-toolbox-3. *Perception*.

[25] Benjamini Y., Hochberg Y. (1995). Controlling the false discovery rate: a practical and powerful approach to multiple testing. *Journal of the Royal Statistical Society: Series B (Methodological)*.

[26] Hughes C., Graham A. (2002). Measuring executive functions in childhood: problems and solutions?. *Child and Adolescent Mental Health*.

[27] Ravaglia G., Forti P., Maioli F. (2003). Homocysteine and cognitive function in healthy elderly community dwellers in Italy. *The American Journal of Clinical Nutrition*.

[28] Faux N. G., Ellis K. A., Porter L. (2011). Homocysteine, vitamin B_12_, and folic acid levels in Alzheimer’s disease, mild cognitive impairment, and healthy elderly: baseline characteristics in subjects of the Australian Imaging Biomarker Lifestyle study. *Journal of Alzheimer's Disease*.

[29] Avants B. B., Tustison N., Song G. (2009). Advanced normalization tools (ANTS). *Insight*.

[30] Cox R. W. (1996). AFNI: software for analysis and visualization of functional magnetic resonance neuroimages. *Computers and Biomedical Research*.

[31] Jenkinson M., Beckmann C. F., Behrens T. E. J., Woolrich M. W., Smith S. M. (2012). FSL. *NeuroImage*.

[32] Seeley W. W., Menon V., Schatzberg A. F. (2007). Dissociable intrinsic connectivity networks for salience processing and executive control. *Journal of Neuroscience*.

[33] Reynolds E. (2006). Vitamin B_12_, folic acid, and the nervous system. *The Lancet Neurology*.

[34] Vogiatzoglou A., Refsum H., Johnston C. (2008). Vitamin B_12_ status and rate of brain volume loss in community-dwelling elderly. *Neurology*.

[35] de Lau L. M. L., Smith A. D., Refsum H., Johnston C., Breteler M. M. B. (2009). Plasma vitamin B_12_ status and cerebral white-matter lesions. *Journal of Neurology, Neurosurgery & Psychiatry*.

[36] Gupta P. K., Gupta R. K., Garg R. K. (2014). DTI correlates of cognition in conventional MRI of normal-appearing brain in patients with clinical features of subacute combined degeneration and biochemically proven vitamin B_12_ deficiency. *American Journal of Neuroradiology*.

[37] Uddin L. Q., Supekar K. S., Ryali S., Menon V. (2011). Dynamic reconfiguration of structural and functional connectivity across core neurocognitive brain networks with development. *Journal of Neuroscience*.

[38] Di X., Biswal B. B. (2014). Modulatory interactions between the default mode network and task positive networks in resting-state. *PeerJ*.

[39] Yarkoni T., Poldrack R. A., Nichols T. E., Van Essen D. C., Wager T. D. (2011). Large-scale automated synthesis of human functional neuroimaging data. *Nature Methods*.

[40] Bonnelle V., Ham T. E., Leech R. (2012). Salience network integrity predicts default mode network function after traumatic brain injury. *Proceedings of the National Academy of Sciences of the United States of America*.

[41] Sharp D. J., Beckmann C. F., Greenwood R. (2011). Default mode network functional and structural connectivity after traumatic brain injury. *Brain*.

[42] Sridharan D., Levitin D. J., Menon V. (2008). A critical role for the right fronto-insular cortex in switching between central-executive and default-mode networks. *Proceedings of the National Academy of Sciences of the United States of America*.

[43] Rushworth M. F. S. (2008). Intention, choice, and the medial frontal cortex. *Annals of the New York Academy of Sciences*.

[44] Ide J. S., Shenoy P., Yu A. J., Li C. S. R. (2013). Bayesian prediction and evaluation in the anterior cingulate cortex. *Journal of Neuroscience*.

[45] Li C., Zheng J., Wang J., Gui L., Li C. (2009). An fMRI stroop task study of prefrontal cortical function in normal aging, mild cognitive impairment, and Alzheimer’s disease. *Current Alzheimer Research*.

[46] Chand G. B., Wu J., Hajjar I., Qiu D. (2017). Interactions of the salience network and its subsystems with the default-mode and the central-executive networks in normal aging and mild cognitive impairment. *Brain Connectivity*.

[47] Vogel J. J., Bowers C. A., Vogel D. S. (2003). Cerebral lateralization of spatial abilities: a meta-analysis. *Brain and Cognition*.

[48] Doricchi F., Thiebautdeschotten M., Tomaiuolo F., Bartolomeo P. (2008). White matter (dis)connections and gray matter (dys)functions in visual neglect: gaining insights into the brain networks of spatial awareness. *Cortex*.

[49] Danti S., Handjaras G., Cecchetti L., Beuzeron-Mangina H., Pietrini P., Ricciardi E. (2018). Different levels of visual perceptual skills are associated with specific modifications in functional connectivity and global efficiency. *International Journal of Psychophysiology*.

[50] Gur R. C., Alsop D., Glahn D. (2000). An fMRI study of sex differences in regional activation to a verbal and a spatial task. *Brain and Language*.

[51] Lütcke H., Frahm J. (2008). Lateralized anterior cingulate function during error processing and conflict monitoring as revealed by high-resolution fMRI. *Cerebral Cortex*.

[52] Stephan K. E., Marshall J. C., Friston K. J. (2003). Lateralized cognitive processes and lateralized task control in the human brain. *Science*.

[53] Tracey I. (2011). Can neuroimaging studies identify pain endophenotypes in humans?. *Nature Reviews Neurology*.

