# Supplementary material for: Brain Hemodynamic Intermediate Phenotype Links Vitamin B12 to Cognitive Profile of Healthy and Mild Cognitive Impaired Subjects
Source: Neural Plast. 2019 Jun 2;2019:6874805. doi: 10.1155/2019/6874805 (PMC6589271; doi:10.1155/2019/6874805)
Supplement: Supplementary 1 — Supplementary Methods: a paragraph detailing the neuropsychological assessment, clinical biochemistry evaluation, and MRI data analysis. Supplementary Results: all the results that are not included in the main manuscript. Appendix: list of the members and affiliations of the Train the Brain Consortium. Supplementary Figure 1: the flowchart for the selection of participants. Supplementary Figure 2: the stimuli employed in the fMRI experiment. Supplementary Figure 3: additional results for the correlation between brain activity and blood biochemistry markers. Supplementary Figure 4: resulting model for the relationship between blood biochemistry, right vmPFC activity, and cognitive profiles. Supplementary Figure 5: resulting model for the relationship between blood biochemistry, bilateral vmPFC activity, and cognitive profiles. Supplementary References. Supplementary Table 1: demographic and neuropsychological data. Supplementary Table 2: predictive power of biochemical markers on cognitive profiles. Supplementary Table 3: predictive power of right dACC activity on cognitive profiles. Supplementary Table 4: predictive power of vmPFC activity on cognitive profiles. Supplementary Table 5: predictive power of right dACC activity on cognitive profiles, controlling for vitamin B12. Supplementary Table 6: predictive power of vmPFC activity on cognitive profiles, controlling for blood biochemistry. Supplementary Table 7: predictive power of fMRI task accuracy on cognitive profiles. [file 6874805.f1.pdf]

Supplementary Methods

Neuropsychological Assessment. Mini-Mental State Examination (1) was adopted to measure subjects' global cognitive efficiency, whilst Raven's Progressive Matrices (2) evaluated nonverbal reasoning abilities and fluid intelligence. In addition, the Rey Auditory Verbal Learning Test (3) and the Babcock Story Recall Test (4) assessed immediate and delayed verbal memory; the Forward Digit Span (5), the Attentional Matrices Test (6) and the Trail Making Test A and B (7) were used to measure attention, visual search, working memory storage capacity and task-switching abilities; the Rey–Osterrieth complex figure (8) immediate and delayed recall, as well as the Corsi Block-Tapping Test (9) for visuo-spatial memory performance. The Freehand Drawing Test (6), the Planned Copy Test (10) and the Rey–Osterrieth complex figure freehand copy (8) assessed subjects' praxis abilities. Lastly, the Phonemic and Semantic Fluency Tests (6) measured vocabulary size and lexical access speed, which are two relevant components of language and executive functions.

Clinical Biochemistry Evaluation. We used a General Linear Model (GLM) to estimate the association between tHcy, folate, vitamin B_{12} as well as their interactions, and age, including years of education, MMSE scores and fMRI task accuracy as nuisance variables. The same method was employed to test linear relationships between blood biochemistry and subjects' global cognitive status (i.e., MMSE scores). In addition, six independent GLMs were adopted to investigate the differences between healthy elders (HE) and mild cognitive impairment (MCI) participants for each vitamin-related compound (and relative interactions), adding age, education and MMSE scores as regressors of no interest.

fMRI Task Accuracy. GLM analysis was employed to investigate differences between HE and MCI subjects in task accuracy during fMRI acquisition, adding age, years of education and MMSE scores in the model as regressors of no interest (p-value < 0.05). The same approach was also adopted to test relationships between fMRI task accuracy, blood biochemistry, age, years of education and MMSE scores.

MRI Data Analysis. High-resolution structural images were used to compute the transformation from the original to the MNI152 standard space (11) and to define anatomically accurate cerebrospinal fluid (CSF) masks. These masks were used to extract signals of no-interest from subjects' fMRI data that were included as confounds when estimating activation maps at single-subject level. To do so, we first used ANTs to perform accurate brain extraction (antsBrainExtraction.sh - https://github.com/stnava/ANTs/blob/master/Scripts/antsBrainExtraction.sh) and AFNI's 3dQwarp to non-linearly transform each brain to match the MNI152 template (1x1x1mm resolution). The estimated deformation field was subsequently used to transform single-subject level activation maps from the original into the standard space. Brain extracted images were also segmented into 3 matters (grey, white and CSF) by means of FSL-FAST. Afterwards, CSF probability maps were thresholded and binarized using the stringent value of 100%. The resulting masks were then eroded by 2 voxels and resampled to match the fMRI spatial grid using nearest-neighbour interpolation. The whole procedure ensured that only few and accurately selected voxels formed the extraction masks.

For each subject, the three functional datasets were pre-processed to remove potential confounds related to physiological artifacts and to reduce scanner-related noise before estimating the statistical parametric maps. First, raw data were automatically inspected and corrected for intensity spikes (3dDespike). Second, images were

adjusted for slice acquisition timing accordingly to the ascending interleaved pattern (3dTshift), and head movements were estimated and compensated (3dvolreg) by registering each volume to the last of the third run (i.e., the closest in time to the high-resolution anatomical sequence). To this regard, a rigid body transformation was adopted and the six estimated motion parameters (x- y- and z- rotations and translations) were included as confounds in the single-subject GLM analysis. In addition, transformation matrices were used to compute an aggregated measure - framewise displacement (FD; fsl_motion_outliers, 12) - that highlighted timepoints affected by excessive motion. We then thresholded this metric (FD ≥ 0.5) to generate a regressor for each motion spike, which was then added as confound to the GLM (i.e., spike regression method, 13). Based on the FD metric we excluded four subjects from the group-level analysis, since their FD across three runs exceeded the group average by 3 standard deviations or more (FD group average = 0.158 ± 0.074; range: 0.069 - 0.435).

Subsequently, time-series were spatially smoothed using a Gaussian kernel until data reached a full width at half maximum level of 6mm (3dBlurToFWHM), so as the original data smoothness was properly considered. Lastly, each fMRI run was normalized (3dTstat and 3dcalc), meaning that for each voxel the average signal across timepoints was 100 and changes between rest and task periods could be expressed as percentage of increments or decrements. Pre-processed and normalized runs were then fed into the single-subject GLM analysis. To estimate activation maps elicited by the visuo-spatial attention task, we adopted a mass-univariate (i.e., voxel-wise) GLM approach, including brain activity as the explained variable and the task regressor, convolved with a canonical hemodynamic response function (SPMG1 in 3dDeconvolve), as the explanatory variable of interest. A 2nd order degree

polynomial regressor (automatically estimated by AFNI), together with the aforementioned six motion parameters, spike regressors and average CSF signal were included in the model as nuisance variables. Temporal autocorrelation of the fMRI signal was also removed using restricted maximum likelihood method with ARMA(1,1) model (3dREMLfit). Task-related changes in hemodynamic activity were estimated as parameter fitting for the regressor of interest (i.e., β-values) and were transformed into T-values - i.e., β/SE(β). GLM residuals were also employed to compute the parameters for the spatial autocorrelation function (3dFWHMx) after being transformed to match the MNI152 standard space and were then used to properly correct group-level results with the AFNI's cluster-based method.

Both β- and T-values were transformed into the MNI152 standard space as well, by applying the already computed deformation field and resampled to a final grid of 2x2x2 mm using sinc interpolation (5mm window). Preprocessed single-subject data were then fed into a group-level correlation analyses to test the relationship between brain activity, blood biochemistry and neuropsychological profiles.

Supplementary Results

Neuropsychological Assessment. Subjects' performance at the immediate recall for Rey Auditory Verbal Learning Test showed a strong correlation with delayed recall of the same test, Trail Making A and B, Rey–Osterrieth complex figure recall, Babcock Story Recall Test and both Phonemic and Semantic Fluency Tests. The delayed recall for Rey Auditory Verbal Learning Test significantly correlated with Corsi Block-Tapping Test, while Attentional matrices with both the Corsi Block-Tapping Test and the Trail Making A and B. Subjects' scores for the latters were associated with Semantic Fluency and both verbal fluency tests (i.e., Phonemic and Semantic), respectively. Both Trail Making A and B tests were correlated also with Raven Progressive Matrices, performance at the Rey–Osterrieth complex figure copy and between themselves. In addition, copy of the Rey–Osterrieth complex figure was associated to Raven Progressive Matrices, Planned Copy and Drawing and Copying Test. On the other hand, both the immediate and delayed recall of the complex figure were correlated with Semantic Fluency, Raven Progressive Matrices, the delayed recall of the Babcock Story and between themselves as well. In our sample, immediate recall performance at the Babcock Story was associated to the delayed recall of the same test, while the Drawing and Copying Test was correlated with Planned Copy and Raven Progressive Matrices. The latter had a linear relationship with Planned Copy and Semantic Fluency. Lastly, scores at the two verbal fluency tests were significantly correlated.

Clinical Biochemistry Evaluation. Overall, vitamin-related compounds were not associated with age (tHcy ~ age: $\beta = 0.202$, p-value = 0.107; folate ~ age: $\beta = 0.167$, p-value = 0.185; vitamin B_{12} ~ age: $\beta = -0.131$, p-value = 0.297; vitamin B_{12} * folate ~ age: $\beta = -0.095$, p-value = 0.453; tHcy * folate ~ age: $\beta = -0.058$, p-value = 0.644).

However, the correlation between the vitamin B_{12} * tHcy interaction and age reached $\beta = 0.272$ (p-value = 0.028).

In addition, MMSE scores were not associated with any of the following blood markers: MMSE scores ~ tHcy: $\beta = -0.100$, p-value = 0.429; MMSE scores ~ folate: $\beta = -0.109$, p-value = 0.390; MMSE scores ~ vitamin B_{12}: $\beta = -0.005$, p-value = 0.972; MMSE scores ~ vitamin B_{12} * folate: $\beta = -0.014$, p-value = 0.909; MMSE scores ~ tHcy * folate: $\beta = -0.158$, p-value = 0.210. Yet, the correlation between MMSE scores and vitamin B_{12} * tHcy achieved $\beta = -0.260$ (p-value = 0.036).

Differences in vitamin B_{12} serum levels between HE (468.87 ± 154.44 pg/mL) and MCI (380.12 ± 138.02 pg/mL) attained p = 0.017 ($F_{(1,60)} = 6.081$). Group comparisons for tHcy, folate, vitamin B_{12} * folate, vitamin B_{12} * tHcy and folate * tHcy did not reach the statistical significance level (respectively: $F_{(1,60)} = 0.143$, p-value = 0.707; $F_{(1,60)} = 0.022$, p-value = 0.881; $F_{(1,60)} = 0.189$, p-value = 0.665; $F_{(1,60)} = 1.003$, p-value = 0.321; $F_{(1,60)} = 0.399$, p-value = 0.530).

fMRI Task Accuracy. There was no significant difference ($F_{(1,60)} = 0.531$, p-value = 0.469) in accuracy between HE (80.70% ± 9%) and MCI subjects (80.15% ± 8%).

GLM analysis investigating associations between subjects' performance at the fMRI visuo-spatial attention task and blood biochemistry, age, years of education and MMSE scores, revealed two significant results: subjects' performance was positively associated to the vitamin B_{12} * tHcy interaction scores ($\beta = 0.335$; p-value = 0.006) and negatively associated to age ($\beta = -0.305$; p-value = 0.014). No other significant results were found (accuracy ~ tHcy: $\beta = 0.141$, p-value = 0.263; accuracy ~ folate: $\beta = 0.116$, p-value = 0.356; accuracy ~ vitamin B_{12}: $\beta = -0.164$, p-value = 0.191; accuracy ~ vitamin B_{12} * folate: $\beta = 0.152$, p-value = 0.227; accuracy ~ tHcy * folate:

β = -0.126, p-value = 0.316; accuracy ~ years of education: β = 0.224, p-value = 0.073; accuracy ~ MMSE scores: β = 0.189, p-value = 0.133).

Relationship between task-negative brain regions, blood biochemistry and cognitive profiles. Two ROIs pertaining to the task-negative network and located within the ventromedial prefrontal cortex (vmPFC) were found to negatively correlate with biochemical markers interactions: right vmPFC with B_{12} * folate (β = -0.686; Supplemental Figure 3A; Supplemental Figure 4B), and bilateral vmPFC with tHcy * folate (β = -0.579; Supplemental Figure 3B; Supplemental Figure 5B). Activity of the right vmPFC was able to predict subjects' cognitive state as expressed by the 12th PC (Supplemental Table 4 and Supplemental Figure 4B). Nonetheless, it should be noted that B_{12} * folate levels per se were already sufficient to explain this cognitive profile (Table 1 and Supplemental Figure 4A). Lastly, hemodynamic of the bilateral vmPFC cluster did not correlate with any of the PCs (Supplemental Table 4 and Supplemental Figure 5B). Both these regions overlap the anterior component of the Default Mode Network (14), and their activity is generally negative (i.e., below baseline) during externally focused attention tasks (15). Of these two clusters, we found that only the right vmPFC significantly predicted subjects' cognitive profile: elders demonstrating higher visual working memory abilities (and lower verbal fluency) have a reduced deactivation during task execution, lower folate concentrations and a valuable imbalance between B_{12} and folate serum levels. Nevertheless, this cognitive profile can be explained simply by looking at vitamin-related compounds (Table 1), thus indicating that there is no valuable role of vmPFC as an intermediate phenotype in the prediction of elders' mental abilities (Supplemental Figure 4). This is also corroborated by the results of a control analysis: the inclusion of B_{12} * folate values as confound in the correlation analysis between right vmPFC and cognitive profile

determined a drop in their association ($\beta = 0.284$, raw p-value = 0.0298; Bonferroni corrected p-value = 0.507; Supplemental Table 6). Hence, vitamin-related compounds and vmPFC share similar predictive power in explaining elders' cognitive state.

Appendix

Train the Brain Consortium

L. Maffei[1], E. Picano[2], M.G. Andreassi[2], A. Angelucci[1], F. Baldacci[3], L. Baroncelli[1], T. Begenisic[1], P.F. Bellinvia[2], N. Berardi[1], L. Biagi[4], J. Bonaccorsi[1], E. Bonanni[3], U. Bonuccelli[3], A. Borghini[2], C. Braschi[1], M. Broccardi[1], R.M. Bruno[2,6], M. Caleo[1], C. Carlesi[1,3], L. Carnicelli[3], G. Cartoni[3], M.C. Cenni[1], R. Ceravolo[3], L. Chico[3], S. Cintoli[1,3], G. Cioni[4,6], M. Coscia[1], M. Costa[1], G. D'Angelo[3], P. D'Ascanio[7], M. De Nes[2], S. Del Turco[2], E. Di Coscio[7], M. Di Galante[7], N. di Lascio[2], F. Faita[2], I. Falorni[1,3], U. Faraguna[4,7], A. Fenu[2], L. Fortunato[2], R. Franco[1], L. Gargani[2], R. Gargiulo[1], L. Ghiadoni[3], F.S. Giorgi[3], R. Iannarella[3], C. Iofrida[5], C. Kusmic[2], F. Limongi[1], M. Maestri[3], M. Maffei[2,6], S. Maggi[1], M. Mainardi[1], L. Mammana[1], A. Marabotti[3], V. Mariotti[7], E. Melissari[5], A. Mercuri[2], S. Micera[8,9], S. Molinaro[2], R. Narducci[1], T. Navarra[2], M. Noale[1], C. Pagni[3], S. Palumbo[5], R. Pasquariello[4], T. Pizzorusso[1], A. Poli[1], L. Pratali[2], A. Retico[10], G. Rota[5], A. Sale[1], S. Sbrana[2], G. Scabia[6], M. Scali[1], D. Scelfo[4], R. Sicari[2], G. Siciliano[3], F. Stea[2], S. Taddei[6], G. Tognoni[3], A. Tonacci[2], M. Tosetti[4], S. Turchi[2], L. Volpi.[1,3]

[1] Neuroscience Institute, CNR, Via G. Moruzzi 1, 56124 Pisa, Italy.
[2] Institute of Clinical Physiology of the CNR, Via G. Moruzzi 1, 56100 Pisa, Italy.
[3] Department of Clinical and Experimental Medicine-Neurology Unit, University of Pisa & AOU Pisa, Italy.
[4] IRCCS Stella Maris, Viale del Tirreno 341, Calambrone, Italy.
[5] Department of Surgical, Medical, Molecular Pathology and of Critical Care, University of Pisa, Via Savi 10, 56126 Pisa, Italy.
[6] Department of Clinical and Experimental Medicine, University of Pisa, Via Savi 10, 56126 Pisa, Italy.
[7] Department of Translational research and New technologies in Medicine and Surgery, University of Pisa, Via Savi 10, 56126 Pisa, Italy.
[8] Bertarelli Foundation Chair in Translational Neuroengineering, Center for Neuroprosthetics and Institute of Bioengineering, Ecole Polytechnique Federale de Lausanne, CH-1015 Lausanne, Switzerland.
[9] Scuola Superiore Sant'Anna, P.za Martiri della Libertà 33, 56127 Pisa, Italy.
[10] National Institute of Nuclear Physics (INFN), Pisa section, Largo B. Pontecorvo, 3, 56127 Pisa, Italy.

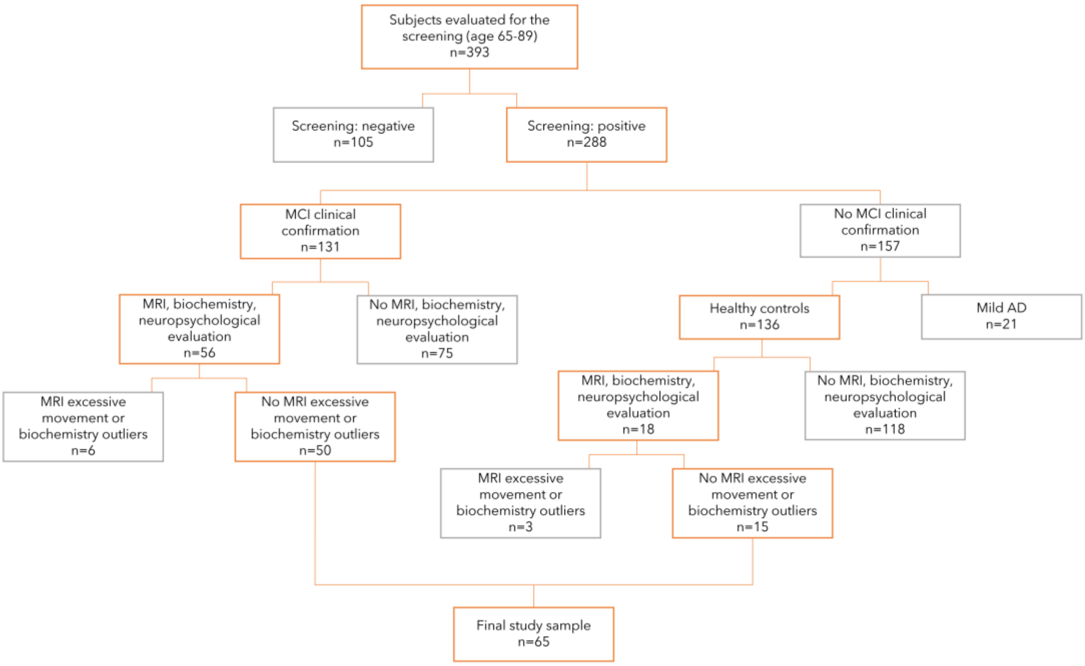

Supplemental Figure 1 **Participants flowchart.** Flowchart representation of the enrolling procedure adopted to reach our final sample of 65 subjects.

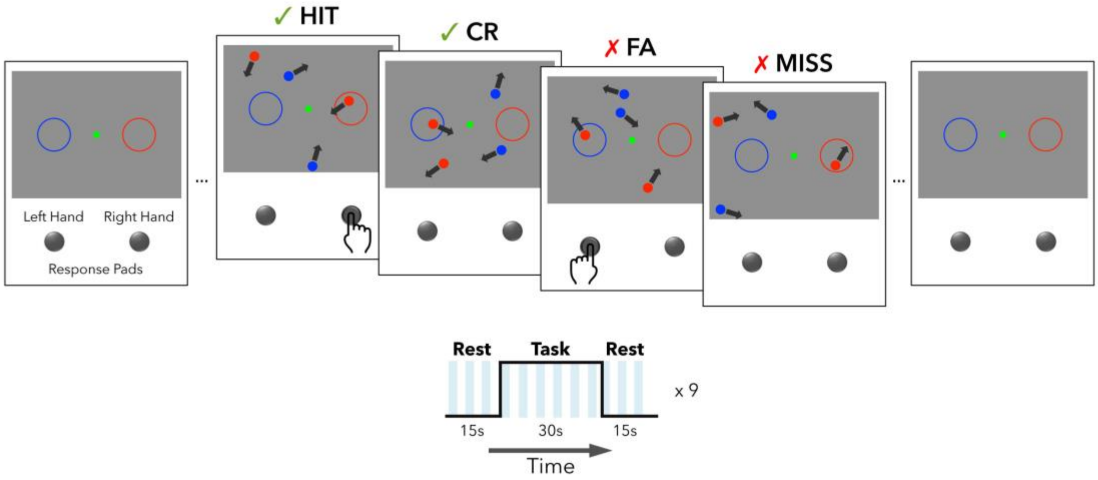

Supplemental Figure 2 **Visuo-spatial attention task.** Schematic representation of the visuo-spatial attention task performed by 65 non-demented elders during fMRI acquisition. Subjects were asked to covertly track four stimuli (i.e., two red and two blue dots) while gazing a central fixation point. The four dots were moving randomly at a constant speed of 6 deg/s. Two near-peripheral (i.e., 7.5 deg of eccentricity) coloured targets were located to the right (i.e., red target) and to the left (i.e., blue target) of the fixation point. Participants had to press the correct response button whenever one stimulus hit the target of the same colour and their performance was recorded in real-time. During rest periods, only the two static targets were presented and subjects were instructed to simply gaze the central fixation point.

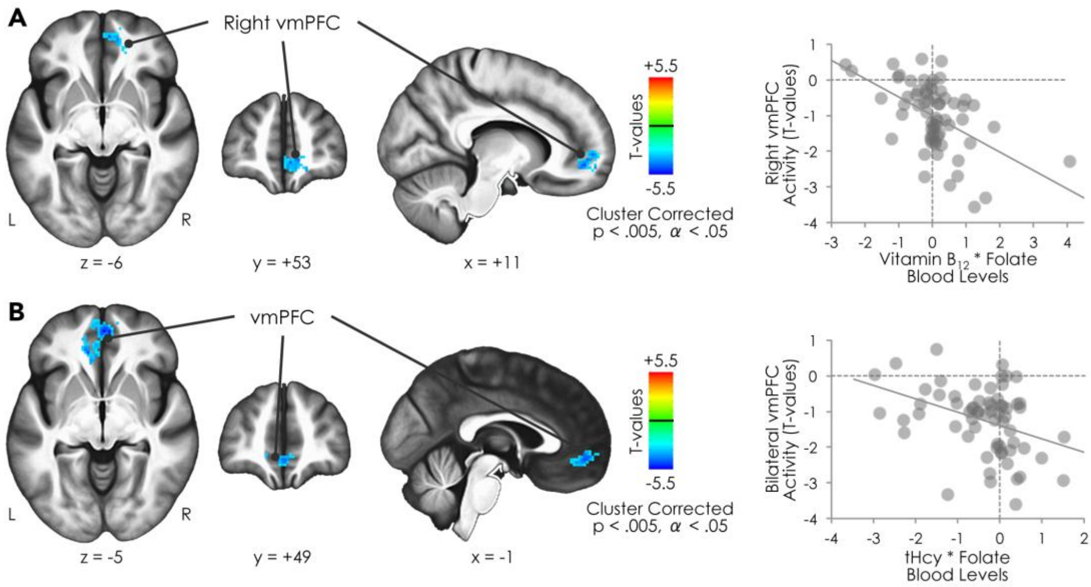

Supplemental Figure 3 **Brain activity results.** (**A**) Results for the relationship between blood biochemistry and task-evoked brain activity. The hemodynamic activity of the right ventromedial prefrontal cortex (vmPFC), located within the task-negative network, was found to be significantly associated with B_{12} * folate interaction (two-tailed p-value < 0.005 for the cluster-forming threshold and α < 0.05 for cluster significance). (**B**) Results for the relationship between blood biochemistry and task-evoked brain activity. The hemodynamic activity of the bilateral vmPFC was found to be significantly associated with homocysteine (tHcy) * folate interaction (two-tailed p-value < 0.005 for the cluster-forming threshold and α < 0.05 for cluster significance).

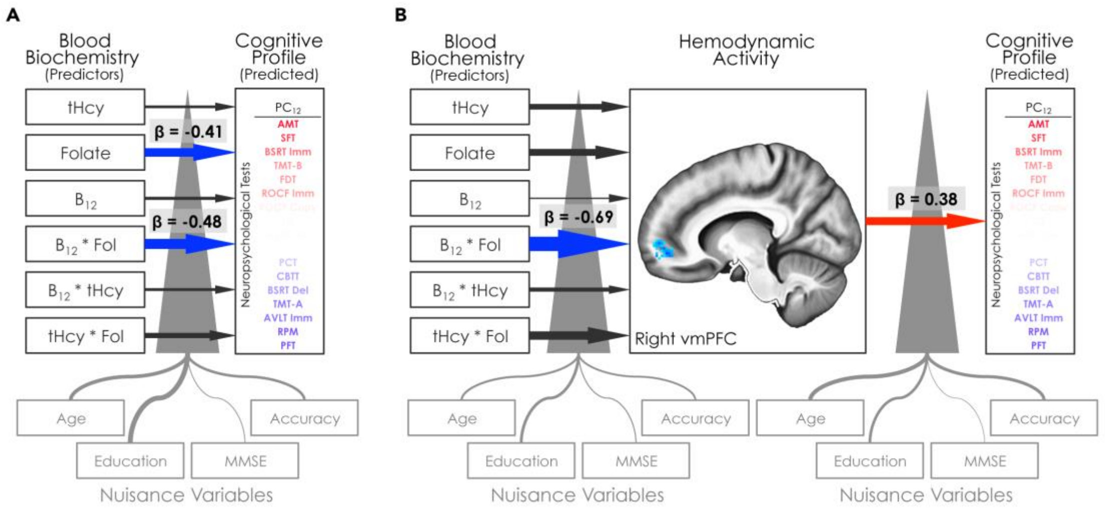

Supplemental Figure 4 **Resulting model for the relationship between blood biochemistry, right vmPFC activity and cognitive profiles**. (**A**) Scheme depicting the significant role of folate (β = -0.41) and B12 by folate interaction (β = -0.48) as direct predictors of the cognitive profile expressed by the 12th component (PC; i.e., visual working memory abilities). Loadings of each neuropsychological test for this component are colour-coded: the more intense the red is, the more positive is the loading of a test; vice-versa, the more intense the blue is, the more negative the loading. Age, years of education, Mini Mental State Examination scores and fMRI task accuracy are included in the model as nuisance variables. (**B**) Hemodynamic activity of the right ventromedial prefrontal cortex (vmPFC) correlated with B12 * folate interaction (β = -0.69) and predicted subjects' cognitive status expressed by the 12th component (β = 0.38). In both panels, arrows thickness represents the strength of the association between variables. Significant correlations are marked with coloured arrows (positive relationships red, negative blue). The same nuisance variables are included in the model at both steps of analysis. Please note that although the right vmPFC was significantly associated to cognitive profile expressed by the 12th PC, hemodynamic activity of this region did not add a relevant contribution to the prediction of subjects' working-memory abilities, since the latter could be already explained by considering blood biochemistry only.

RPM: Raven's Progressive Matrices, AVLT: Rey Auditory Verbal Learning Test, BSRT: Babcock Story Recall Test, DS: Forward Digit Span, AMT: Attentional Matrices Test, TMT-: Trail Making Test, ROCF: Rey–Osterrieth complex figure, CBTT: Corsi Block-Tapping Test, FDT: Freehand Drawing Test, PCT: Planned Copy Test, PFT: Phonemic Fluency Test, SFT: Semantic Fluency Test. Imm: immediate

recall, Del: delayed recall. tHcy: homocysteine, Fol: folate, MMSE: Mini-Mental State Examination.

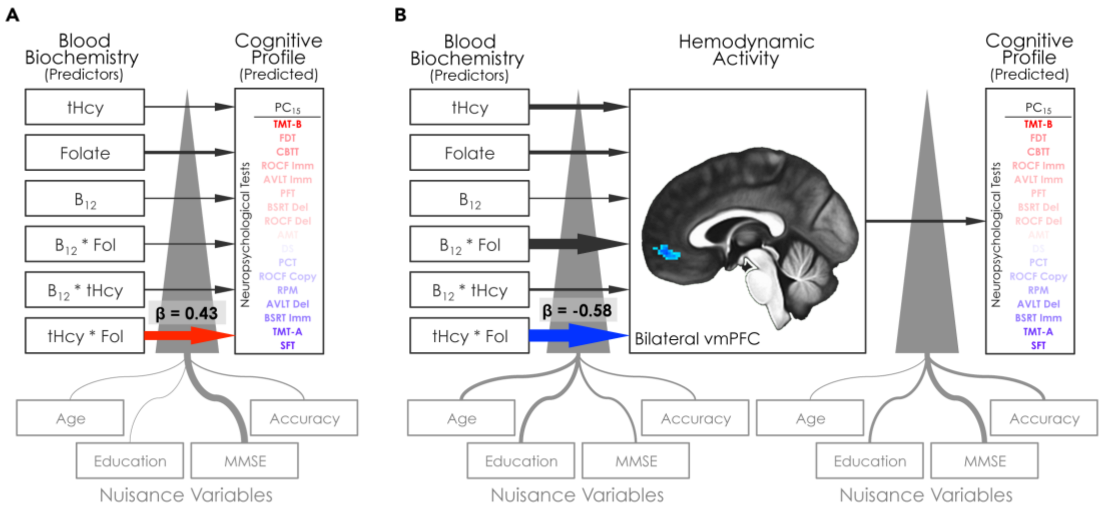

Supplemental Figure 5 **Resulting model for the relationship between blood biochemistry, bilateral vmPFC activity and cognitive profiles**. (**A**) Scheme depicting the significant role of homocysteine by folate interaction (β = 0.43), as direct predictor of the cognitive profile expressed by the 15th component (PC; i.e., task switching and attention abilities). Loadings of each neuropsychological test for this component are colour-coded: the more intense the red is, the more positive is the loading of a test; vice-versa, the more intense the blue is, the more negative the loading. Age, years of education, Mini Mental State Examination scores and fMRI task accuracy are included in the model as nuisance variables. (**B**) Hemodynamic activity of the bilateral ventromedial prefrontal cortex (vmPFC) correlated with tHcy * folate interaction (β = -0.58), yet it failed to predict subjects' cognitive status expressed by the 15th component. In both panels, arrows thickness represents the strength of the association between variables. Significant correlations are marked with coloured arrows (positive relationships red, negative blue). The same nuisance variables are included in the model at both steps of analysis.

RPM: Raven's Progressive Matrices, AVLT: Rey Auditory Verbal Learning Test, BSRT: Babcock Story Recall Test, DS: Forward Digit Span, AMT: Attentional Matrices Test, TMT-: Trail Making Test, ROCF: Rey–Osterrieth complex figure, CBTT: Corsi Block-Tapping Test, FDT: Freehand Drawing Test, PCT: Planned Copy Test, PFT: Phonemic Fluency Test, SFT: Semantic Fluency Test. Imm: immediate recall, Del: delayed recall. tHcy: homocysteine, Fol: folate, MMSE: Mini-Mental State Examination.

References

1. Folstein MF, Folstein SE, McHugh PR. “Mini-mental state”: a practical method for grading the cognitive state of patients for the clinician. J Psychiatr Res 1975;12(3):189–98.

2. Raven JC. Mental tests used in genetic studies: the performance of related individuals on tests mainly educative and mainly reproductive. University of London Unpublished Master’s thesis 1936.

3. Rey A. Auditory-Verbal Learning Test (AVLT). Press Universitaire de France 1964.

4. Babcock H, Levy L. Test and manual of directions; the revised examination for the measurement of efficiency of mental functioning. CH Stoelting 1940.

5. Wechsler D. A standardized memory scale for clinical use. The Journal of Psychology 1945;19.1:87–95.

6. Spinnler H, Tognoni G. Italian group on the neuropsychological study of ageing: Italian standardization and classification of neuropsychological tests. Ital J Neurol Sci 1987;6(suppl 8):1–120.

7. Reitan RM. Vailidity of the Trail Making Test as an indicator of organic brain damage. Perceptual and motor skills 1958;8(3):271–6.

8. Rey A. L’examen psychologique dans les cas d’encéphalopathie traumatique (Les problems). Archives de psychologie 1941;28:215–85.

9. Corsi PS. Human memory and the medial temporal lobe of the brain. Unpublished doctoral dissertation, Mc Gill University 1972.

10. Gainotti G, Miceli G, Caltagirone C. Constructional apraxia in left brain-damaged patients: a planning disorder? Cortex 1977;13(2):109–18.

11. Fonov VS, Evans AC, McKinstry RC, Almli CR, Collins DR. Unbiased nonlinear average age-appropriate brain templates from birth to adulthood. NeuroImage 2009;47(s1):s102.

12. Power JD, Barnes KA, Snyder AZ, Schlaggar BL, Petersen SE. Spurious but systematic correlations in functional connectivity MRI networks arise from subject motion. Neuroimage 2012;59(3):2142–54.

13. Satterthwaite TD, Elliott MA, Gerraty RT, Ruparel K, Loughead J, Calkins ME, Eickhoff SB, Hakonarson H, Gur RC, Gur RE, Wolf DH. An improved framework for confound regression and filtering for control of motion artifact in the

preprocessing of resting-state functional connectivity data. Neuroimage 2013;64:240–56.
14. Raichle ME, MacLeod AM, Snyder AZ, Powers WJ, Gusnard DA, Shulman GL. A default mode of brain function. Proc Natl Acad Sci 2001;98(2):676–82.
15. Gusnard DA, Raichle ME. Searching for a baseline: functional imaging and the resting human brain. Nat Rev Neurosci 2001;2(10):685–94.

Supplemental Table 1: Demographic data and neuropsychological scores

Subject ID	Gender	Condition	Age		Education		MMSE		AVLT Imm		AVLT Del		DS		AMT		TMT-A*		TMT-B*	
			Score	Z-score	Score	Z-score	Score	Z-score	Score	Z-score	Score	Z-score	Score	Z-score	Score	Z-score	Score	Z-score	Score	Z-score
sub-0001	M	aMCI	79	0.97	15	1.08	26.3	0.20	34.6	0.19	4.4	-0.27	6.75	1.24	55.50	0.92	47	-0.05	92	0.53
sub-0002	F	aMCI	72	-0.40	13	0.64	24.7	-0.58	15.2	-1.90	1.9	-1.09	5.75	0.25	58.75	1.38	15	0.95	128	0.15
sub-0003	M	aMCI	75	0.19	8	-0.45	24.0	-0.93	22.9	-1.07	2.6	-0.86	4.25	-1.23	45.00	-0.57	24	0.67	116	0.27
sub-0004	M	aMCIm	70	-0.80	13	0.64	23.7	-1.07	28.1	-0.51	1.2	-1.32	4.75	-0.74	51.75	0.39	84	-1.20	144	-0.03
sub-0005	M	aMCI	69	-0.99	5	-1.11	28.3	1.18	22.0	-1.17	3.4	-0.60	5.50	0.00	44.00	-0.71	36	0.29	134	0.08
sub-0006	F	aMCI	73	-0.21	8	-0.45	26.4	0.25	39.9	0.76	6.6	0.44	6.25	0.75	57.50	1.21	52	-0.21	113	0.31
sub-0007	M	aMCI	73	-0.21	13	0.64	26.7	0.40	35.1	0.25	4.9	-0.11	5.75	0.25	39.25	-1.39	20	0.79	83	0.63
sub-0008	F	aMCIm	81	1.37	8	-0.45	23.7	-1.07	21.1	-1.27	3.3	-0.63	4.25	-1.23	45.00	-0.57	134	-2.76	283	-1.52
sub-0009	M	aMCI	77	0.58	13	0.64	26.3	0.20	35.4	0.28	5.6	0.12	7.00	1.49	58.50	1.35	21	0.76	50	0.98
sub-0010	F	aMCIm	67	-1.39	5	-1.11	21.9	-1.95	22.0	-1.17	5.8	0.18	4.50	-0.99	50.50	0.21	113	-2.11	283	-1.52
sub-0011	M	aMCI	69	-0.99	8	-0.45	27.0	0.54	36.9	0.44	3.9	-0.44	7.25	1.73	55.50	0.92	2	1.35	0	1.52
sub-0012	F	HE	69	-0.99	5	-1.11	27.9	0.98	44.1	1.22	8.8	1.16	5.50	0.00	51.75	0.39	29	0.51	59	0.89
sub-0013	M	aMCIm	74	-0.01	5	-1.11	24.7	-0.58	28.0	-0.52	4.1	-0.37	3.50	-1.97	39.25	-1.39	66	-0.64	283	-1.52
sub-0014	F	aMCIm	73	-0.21	5	-1.11	22.3	-1.76	25.0	-0.85	0.0	-1.71	5.50	0.00	40.75	-1.17	42	0.11	283	-1.52
sub-0015	F	HE	71	-0.60	18	1.74	23.7	-1.07	40.3	0.81	6.8	0.51	6.50	0.99	57.75	1.24	45	0.01	175	-0.36
sub-0016	M	HE	68	-1.19	13	0.64	27.2	0.64	33.1	0.03	5.2	-0.01	4.50	-0.99	55.75	0.96	29	0.51	46	1.03
sub-0017	F	HE	78	0.78	8	-0.45	27.0	0.54	36.1	0.35	9.3	1.33	5.25	-0.24	38.00	-1.56	11	1.07	159	-0.19
sub-0018	F	aMCI	74	-0.01	13	0.64	27.7	0.88	42.2	1.01	10.9	1.85	3.75	-1.73	48.75	-0.04	36	0.29	148	-0.07
sub-0019	M	aMCIm	83	1.76	5	-1.11	27.4	0.74	27.2	-0.61	6.8	0.51	5.75	0.25	41.25	-1.10	129	-2.61	283	-1.52
sub-0020	F	aMCI	81	1.37	5	-1.11	29.4	1.72	39.2	0.69	7.8	0.84	5.50	0.00	50.25	0.18	38	0.23	128	0.15
sub-0021	M	HE	73	-0.21	8	-0.45	25.4	-0.24	39.9	0.76	7.6	0.77	5.25	-0.24	45.00	-0.57	22	0.73	132	0.10
sub-0022	F	aMCI	71	-0.60	8	-0.45	23.4	-1.22	32.0	-0.09	3.4	-0.60	9.25	3.71	43.50	-0.78	57	-0.36	170	-0.31
sub-0023	F	HE	80	1.17	14	0.86	26.3	0.20	40.7	0.85	10.3	1.65	6.00	0.50	52.00	0.42	20	0.79	37	1.12
sub-0024	M	aMCI	69	-0.99	5	-1.11	24.3	-0.78	21.0	-1.28	2.4	-0.93	4.50	-0.99	53.00	0.57	27	0.57	35	1.14
sub-0025	M	aMCI	74	-0.01	18	1.74	25.7	-0.09	32.3	-0.06	1.4	-1.25	5.50	0.00	44.25	-0.68	70	-0.77	122	0.21
sub-0026	F	HE	76	0.38	8	-0.45	27.0	0.54	50.9	1.95	8.6	1.10	4.25	-1.23	55.00	0.85	18	0.85	13	1.38
sub-0027	F	HE	73	-0.21	5	-1.11	29.3	1.67	38.0	0.56	8.1	0.93	6.50	0.99	51.75	0.39	31	0.45	112	0.32
sub-0028	M	aMCIm	64	-1.98	13	0.64	27.2	0.64	23.0	-1.06	2.6	-0.86	4.25	-1.23	56.25	1.03	39	0.20	60	0.88
sub-0029	F	naMCIm	82	1.56	17	1.52	27.1	0.59	30.6	-0.24	5.2	-0.01	4.75	-0.74	34.00	-2.13	156	-3.45	283	-1.52
sub-0030	F	aMCIm	84	1.96	5	-1.11	22.4	-1.71	19.2	-1.47	4.8	-0.14	6.75	1.24	45.75	-0.46	35	0.32	283	-1.52
sub-0031	M	HE	74	-0.01	17	1.52	22.7	-1.56	44.3	1.24	8.8	1.16	5.50	0.00	43.75	-0.75	53	-0.24	104	0.40
sub-0032	M	naMCI	66	-1.58	17	1.52	27.2	0.64	31.5	-0.14	5.1	-0.05	5.50	0.00	44.25	-0.68	40	0.17	114	0.30
sub-0033	M	HE	77	0.58	8	-0.45	27.0	0.54	38.1	0.57	9.3	1.33	4.25	-1.23	36.50	-1.78	29	0.51	84	0.62
sub-0034	F	naMCI	75	0.19	5	-1.11	28.7	1.37	31.0	-0.20	7.1	0.61	4.50	-0.99	50.75	0.25	33	0.39	127	0.16
sub-0035	F	aMCI	65	-1.78	8	-0.45	26.0	0.05	38.0	0.56	9.3	1.33	5.25	-0.24	47.75	-0.18	32	0.42	283	-1.52
sub-0036	F	HE	72	-0.40	8	-0.45	27.4	0.74	43.9	1.20	7.9	0.87	6.25	0.75	59.00	1.42	4	1.29	25	1.25
sub-0037	F	aMCIm	74	-0.01	5	-1.11	23.3	-1.27	30.0	-0.31	4.1	-0.37	6.50	0.99	50.75	0.25	63	-0.55	283	-1.52
sub-0038	F	HE	81	1.37	13	0.64	29.1	1.57	52.4	2.11	11.6	2.08	6.00	0.50	56.00	0.99	56	-0.33	15	1.36
sub-0039	F	aMCIm	77	0.58	5	-1.11	26.7	0.40	15.2	-1.90	3.8	-0.47	5.50	0.00	52.25	0.46	97	-1.61	283	-1.52
sub-0040	M	HE	72	-0.40	18	1.74	27.7	0.88	50.3	1.89	9.8	1.49	6.50	0.99	51.75	0.39	15	0.95	43	1.06
sub-0041	F	HE	66	-1.58	17	1.52	27.2	0.64	61.5	3.10	13.1	2.57	4.50	-0.99	53.75	0.67	19	0.82	90	0.55
sub-0042	M	HE	67	-1.39	17	1.52	25.2	-0.34	54.5	2.34	12.1	2.24	6.50	0.99	50.25	0.18	37	0.26	92	0.53
sub-0043	F	aMCI	76	0.38	8	-0.45	26.0	0.05	25.9	-0.75	3.6	-0.54	5.25	-0.24	59.50	1.49	31	0.45	37	1.12
sub-0044	F	naMCI	80	1.17	8	-0.45	24.7	-0.58	30.1	-0.29	5.3	0.02	5.25	-0.24	58.00	1.28	32	0.42	283	-1.52
sub-0045	M	aMCIm	80	1.17	8	-0.45	27.7	0.88	28.1	-0.51	3.3	-0.63	6.25	0.75	60.50	1.63	52	-0.21	283	-1.52
sub-0046	F	aMCI	72	-0.40	5	-1.11	26.3	0.20	29.0	-0.41	2.4	-0.93	6.50	0.99	56.50	1.06	34	0.36	92	0.53
sub-0047	F	aMCI	81	1.37	8	-0.45	26.7	0.40	32.1	-0.08	3.3	-0.63	5.25	-0.24	44.00	-0.71	38	0.23	100	0.45
sub-0048	F	aMCI	72	-0.40	11	0.21	26.4	0.25	21.1	-1.27	1.2	-1.32	4.75	-0.74	47.25	-0.25	32	0.42	118	0.25
sub-0049	M	aMCI	70	-0.80	18	1.74	25.7	-0.09	29.3	-0.38	2.8	-0.80	6.50	0.99	42.75	-0.89	41	0.14	68	0.79
sub-0050	M	aMCI	71	-0.60	13	0.64	26.7	0.40	31.1	-0.19	3.2	-0.67	4.75	-0.74	56.75	1.10	30	0.48	15	1.36
sub-0051	F	aMCI	71	-0.60	18	1.74	25.7	-0.09	29.3	-0.38	3.8	-0.47	5.50	0.00	49.75	0.11	38	0.23	78	0.68
sub-0052	F	aMCIm	80	1.17	5	-1.11	24.4	-0.73	28.2	-0.50	4.8	-0.14	3.50	-1.97	33.25	-2.24	129	-2.61	283	-1.52
sub-0053	M	naMCI	78	0.78	5	-1.11	24.7	-0.58	34.2	0.15	6.1	0.28	5.50	0.00	47.75	-0.18	24	0.67	283	-1.52
sub-0054	F	aMCIm	76	0.38	8	-0.45	28.0	1.03	39.9	0.76	4.6	-0.21	4.25	-1.23	54.50	0.78	27	0.57	246	-1.12
sub-0055	M	aMCI	79	0.97	17	1.52	21.3	-2.25	24.6	-0.89	2.2	-0.99	5.75	0.25	39.00	-1.42	57	-0.36	167	-0.27
sub-0056	M	aMCIm	80	1.17	8	-0.45	24.7	-0.58	26.1	-0.73	0.0	-1.71	6.25	0.75	42.50	-0.92	48	-0.08	173	-0.34
sub-0057	F	aMCIm	67	-1.39	8	-0.45	20.0	-2.88	23.0	-1.06	0.0	-1.71	4.25	-1.23	32.75	-2.31	50	-0.14	73	0.74
sub-0058	M	naMCIm	83	1.76	8	-0.45	27.7	0.88	31.1	-0.19	7.3	0.67	5.25	-0.24	41.75	-1.03	83	-1.17	281	-1.50
sub-0059	F	aMCI	67	-1.39	12	0.42	26.2	0.15	35.3	0.27	2.6	-0.86	5.75	0.25	57.75	1.24	4	1.29	47	1.01
sub-0060	M	aMCI	70	-0.80	13	0.64	25.7	-0.09	31.1	-0.19	3.2	-0.67	5.75	0.25	46.75	-0.32	34	0.36	81	0.65
sub-0061	M	aMCI	69	-0.99	13	0.64	25.7	-0.09	26.1	-0.73	3.2	-0.67	4.75	-0.74	51.75	0.39	25	0.64	35	1.14
sub-0062	F	aMCI	73	-0.21	8	-0.45	25.4	-0.24	29.9	-0.32	3.6	-0.54	6.25	0.75	52.50	0.50	40	0.17	118	0.25
sub-0063	M	aMCI	76	0.38	17	1.52	27.3	0.69	39.3	0.70	4.4	-0.27	6.50	0.99	50.75	0.25	43	0.07	108	0.36
sub-0064	F	aMCIm	81	1.37	5	-1.11	23.4	-1.22	31.2	-0.18	3.8	-0.47	6.50	0.99	51.25	0.32	38	0.23	283	-1.52
sub-0065	M	naMCI	73	-0.21	5	-1.11	30.0	2.01	31.0	-0.20	6.1	0.28	5.50	0.00	46.25	-0.39	94	-1.52	168	-0.28
Group avg			74		10		25.9		32.8		5.2		5.5		49.0		45.4		141.5	
Stdev			5		5		2.0		9.3		3.1		1.0		7.0		32.1		93.2	

Subject ID	ROCF Copy		ROCF Imm		ROCF Del		BSRT Imm		BSRT Del		FDT		PCT		RPM		PFT		SFT		CBTT	
	Score	Z-score	Score	Z-score	Score	Z-score	Score	Z-score	Score	Z-score	Score	Z-score	Score	Z-score	Score	Z-score	Score	Z-score	Score	Z-score	Score	Z-score
sub-0001	29.7	-0.08	7.6	-0.85	7.0	-0.81	0.5	-2.10	0.5	-1.37	9.7	-0.58	69.1	0.47	26.8	-0.52	22.3	-1.04	19	-2.10	4.5	-0.23
sub-0002	37.2	1.15	7.3	-0.91	5.4	-1.06	1.5	-1.56	0.5	-1.37	12.4	1.57	69.3	0.54	34.8	1.10	32.5	-0.10	25	-1.36	4.3	-0.60
sub-0003	35.0	0.79	11.9	-0.12	4.3	-1.24	0.4	-2.15	0.7	-1.28	9.8	-0.50	69.3	0.54	32.3	0.59	26.7	-0.63	31	-0.63	4.3	-0.60
sub-0004	18.6	-1.91	4.0	-1.47	4.7	-1.17	3.5	-0.48	3.2	-0.20	6.9	-2.81	64.0	-1.27	25.1	-0.87	10.6	-2.10	36	-0.01	4.0	-0.96
sub-0005	30.8	0.10	9.4	-0.55	14.7	0.40	1.2	-1.72	2.7	-0.42	11.2	0.62	67.3	-0.14	34.5	1.03	35.6	0.18	40	0.48	4.5	-0.23
sub-0006	36.0	0.96	10.1	-0.43	11.7	-0.07	2.5	-1.02	3.9	0.10	9.8	-0.50	71.3	1.22	30.3	0.18	29.7	-0.36	33	-0.38	5.5	1.22
sub-0007	33.7	0.58	9.1	-0.60	9.1	-0.48	3.7	-0.37	3.8	0.06	11.4	0.78	69.3	0.54	31.8	0.49	27.5	-0.56	31	-0.63	5.0	0.49
sub-0008	22.7	-1.23	10.0	-0.44	9.8	-0.37	3.8	-0.31	4.0	0.14	10.9	0.38	69.7	0.68	26.4	-0.61	31.7	-0.18	20	-1.98	2.3	-3.50
sub-0009	33.9	0.61	7.5	-0.87	6.9	-0.83	3.8	-0.31	2.9	-0.33	10.4	-0.02	70.7	1.02	27.5	-0.38	43.5	0.90	37	0.11	5.3	0.86
sub-0010	31.8	0.27	11.0	-0.27	10.7	-0.23	0.2	-2.26	0.7	-1.28	10.2	-0.18	66.7	-0.35	25.5	-0.79	32.6	-0.10	41	0.60	4.3	-0.60
sub-0011	35.4	0.86	23.4	1.85	24.1	1.87	3.5	-0.48	2.5	-0.50	11.6	0.94	67.0	-0.25	34.6	1.06	46.9	1.21	49	1.58	4.3	-0.60
sub-0012	39.3	1.50	24.7	2.07	25.8	2.14	6.4	1.09	7.9	1.82	11.1	0.54	71.5	1.29	29.9	0.10	35.9	0.21	50	1.70	5.5	1.22
sub-0013	37.4	1.19	19.0	1.10	17.3	0.80	5.4	0.55	5.9	0.96	10.3	-0.10	65.1	-0.90	29.2	-0.04	23.4	-0.94	35	-0.14	4.5	-0.23
sub-0014	28.4	-0.30	0.0	-2.16	0.0	-1.91	3.4	-0.53	0.9	-1.19	10.3	-0.10	66.1	-0.55	21.2	-1.66	24.4	-0.84	20	-1.98	4.8	0.13
sub-0015	28.6	-0.26	8.8	-0.65	7.8	-0.69	3.5	-0.48	4.3	0.27	8.4	-1.61	66.3	-0.49	23.4	-1.21	29.5	-0.38	36	-0.01	4.0	-0.96
sub-0016	32.6	0.40	18.7	1.05	12.2	0.00	7.0	1.42	3.2	-0.20	11.9	1.18	68.0	0.10	36.1	1.36	70.6	3.37	54	2.20	5.0	0.49
sub-0017	27.2	-0.49	9.4	-0.55	16.0	0.60	4.8	0.23	5.3	0.70	9.9	-0.42	66.7	-0.35	26.4	-0.61	29.7	-0.36	31	-0.63	4.5	-0.23
sub-0018	26.2	-0.66	15.3	0.46	14.4	0.35	4.8	0.23	1.6	-0.89	9.4	-0.82	68.3	0.20	30.8	0.29	40.5	0.63	38	0.23	4.3	-0.60
sub-0019	34.1	0.64	23.9	1.94	20.9	1.37	2.6	-0.96	4.4	0.32	11.5	0.86	71.5	1.29	30.0	0.12	27.4	-0.57	35	-0.14	5.8	1.58
sub-0020	35.1	0.81	13.6	0.17	13.8	0.26	2.5	-1.02	3.1	-0.24	10.5	0.06	66.5	-0.42	32.0	0.53	48.4	1.35	31	-0.63	4.8	0.13
sub-0021	31.0	0.13	11.4	-0.20	14.8	0.41	3.4	-0.53	5.9	0.96	11.8	1.10	70.3	0.88	29.3	-0.02	41.7	0.73	45	1.09	5.3	0.86
sub-0022	36.8	1.09	12.5	-0.01	10.2	-0.31	3.2	-0.64	0.5	-1.37	8.6	-1.45	64.0	-1.27	24.6	-0.97	37.6	0.36	35	-0.14	4.5	-0.23
sub-0023	34.9	0.78	19.2	1.13	17.9	0.90	5.3	0.50	5.4	0.75	12.4	1.57	70.3	0.88	35.5	1.24	50.5	1.54	48	1.46	4.5	-0.23
sub-0024	34.8	0.76	3.4	-1.57	3.7	-1.33	5.2	0.44	0.7	-1.28	12.2	1.42	70.7	1.02	36.5	1.44	18.6	-1.37	20	-1.98	5.5	1.22
sub-0025	32.0	0.30	3.7	-1.52	3.9	-1.30	4.5	0.06	0.3	-1.45	10.6	0.14	66.1	-0.55	23.1	-1.27	41.3	0.70	40	0.48	3.8	-1.32
sub-0026	35.0	0.79	16.6	0.69	17.7	0.87	6.0	0.87	6.3	1.13	10.8	0.30	70.3	0.88	27.3	-0.42	47.7	1.28	45	1.09	4.5	-0.23
sub-0027	34.4	0.69	26.6	2.40	30.8	2.92	7.0	1.42	7.2	1.52	12.3	1.50	70.1	0.81	38.2	1.78	35.4	0.16	42	0.72	4.8	0.13
sub-0028	35.1	0.81	15.7	0.53	7.8	-0.69	3.1	-0.69	0.0	-1.58	10.8	0.30	65.7	-0.69	33.4	0.81	16.9	-1.53	42	0.72	4.0	-0.96
sub-0029	11.2	-3.13	6.8	-0.99	6.4	-0.91	6.1	0.93	6.1	1.05	9.7	-0.58	63.1	-1.58	14.8	-2.95	17.3	-1.49	24	-1.49	4.3	-0.60
sub-0030	26.1	-0.67	13.6	0.17	13.4	0.19	4.7	0.17	4.5	0.36	9.5	-0.74	66.5	-0.42	31.0	0.33	28.4	-0.48	28	-0.99	4.8	0.13
sub-0031	35.4	0.86	15.4	0.48	15.6	0.54	5.9	0.82	5.7	0.88	11.4	0.78	69.3	0.54	28.4	-0.20	29.5	-0.38	41	0.60	3.8	-1.32
sub-0032	31.9	0.28	14.8	0.38	15.4	0.51	7.8	1.85	7.5	1.65	10.3	-0.10	67.1	-0.21	32.7	0.67	6.8	-2.45	29	-0.87	4.8	0.13
sub-0033	32.2	0.33	16.4	0.65	21.8	1.51	7.4	1.63	7.6	1.69	10.9	0.38	69.7	0.68	32.4	0.61	31.7	-0.18	32	-0.50	4.5	-0.23
sub-0034	22.4	-1.28	18.1	0.94	14.5	0.36	3.4	-0.53	3.9	0.10	8.3	-1.69	68.1	0.13	32.2	0.57	34.4	0.07	40	0.48	4.8	0.13
sub-0035	35.9	0.94	17.2	0.79	18.2	0.95	0.1	-2.31	0.3	-1.45	12.5	1.65	68.7	0.34	30.0	0.12	29.2	-0.41	44	0.97	5.3	0.86
sub-0036	34.4	0.69	15.0	0.41	15.6	0.54	3.8	-0.31	4.1	0.19	10.6	0.14	70.0	0.78	37.6	1.66	37.9	0.39	40	0.48	5.5	1.22
sub-0037	17.4	-2.11	12.1	-0.08	10.3	-0.29	2.4	-1.07	2.9	-0.33	7.3	-2.49	64.1	-1.24	29.2	-0.04	39.4	0.53	30	-0.75	4.8	0.13
sub-0038	34.9	0.78	18.7	1.05	17.9	0.90	7.1	1.47	2.9	-0.33	12.4	1.57	69.7	0.68	35.5	1.24	42.5	0.81	41	0.60	6.3	2.31
sub-0039	21.1	-1.50	10.6	-0.34	10.8	-0.22	3.5	-0.48	4.1	0.19	9.5	-0.74	62.5	-1.78	27.0	-0.48	38.4	0.43	33	-0.38	4.8	0.13
sub-0040	29.4	-0.13	25.1	2.14	25.3	2.06	5.8	0.77	3.3	-0.16	9.4	-0.82	69.3	0.54	30.4	0.20	47.5	1.26	46	1.21	4.8	0.13
sub-0041	31.9	0.28	17.4	0.82	15.9	0.58	7.0	1.42	6.8	1.35	11.3	0.70	69.1	0.47	30.7	0.27	39.8	0.56	40	0.48	4.8	0.13
sub-0042	33.9	0.61	16.8	0.72	18.9	1.06	2.1	-1.23	6.2	1.09	9.3	-0.89	63.1	-1.58	31.7	0.47	35.8	0.20	53	2.07	4.8	0.13
sub-0043	33.0	0.46	14.6	0.34	6.7	-0.86	6.0	0.87	6.0	1.01	9.8	-0.50	68.3	0.20	26.3	-0.63	36.7	0.28	33	-0.38	4.8	0.13
sub-0044	10.2	-3.29	11.5	-0.19	8.8	-0.53	3.8	-0.31	4.0	0.14	8.9	-1.21	70.7	1.02	22.1	-1.48	26.7	-0.63	32	-0.50	5.8	1.58
sub-0045	26.7	-0.58	5.9	-1.14	6.3	-0.92	3.8	-0.31	1.0	-1.15	11.9	1.18	68.7	0.34	25.4	-0.81	22.7	-1.00	34	-0.26	5.5	1.22
sub-0046	28.8	-0.23	11.5	-0.19	13.2	0.16	3.5	-0.48	0.7	-1.28	10.2	-0.18	66.3	-0.49	31.5	0.43	41.6	0.73	40	0.48	5.5	1.22
sub-0047	26.7	-0.58	16.0	0.58	13.3	0.18	3.5	-0.48	1.0	-1.15	11.9	1.18	67.7	-0.01	34.4	1.01	35.7	0.19	36	-0.01	4.5	-0.23
sub-0048	24.6	-0.92	4.0	-1.47	4.7	-1.17	3.5	-0.48	0.2	-1.49	8.9	-1.21	64.0	-1.27	27.1	-0.46	33.6	0.00	27	-1.12	5.3	0.86
sub-0049	27.4	-0.46	10.6	-0.34	5.3	-1.08	5.5	0.60	3.0	-0.29	11.4	0.78	69.2	0.51	30.4	0.20	56.5	2.09	40	0.48	4.0	-0.96
sub-0050	27.6	-0.43	13.0	0.07	9.2	-0.47	5.8	0.77	5.5	0.79	9.9	-0.42	68.0	0.10	29.1	-0.06	29.1	-0.42	25	-1.36	4.0	-0.96
sub-0051	34.4	0.69	10.8	-0.31	11.3	-0.14	5.5	0.60	5.3	0.70	11.4	0.78	69.3	0.54	29.4	0.00	21.5	-1.11	47	1.34	6.0	1.95
sub-0052	37.1	1.14	0.0	-2.16	0.0	-1.91	4.5	0.06	5.1	0.62	11.5	0.86	70.5	0.95	36.0	1.34	28.4	-0.48	31	-0.63	3.8	-1.32
sub-0053	25.1	-0.84	13.4	0.14	11.8	-0.06	6.0	0.87	3.1	-0.24	10.5	0.06	67.5	-0.08	26.0	-0.69	23.4	-0.94	44	0.97	4.5	-0.23
sub-0054	28.0	-0.36	15.1	0.43	16.7	0.71	4.8	0.23	5.9	0.96	8.8	-1.29	66.3	-0.49	28.3	-0.22	39.7	0.55	29	-0.87	4.5	-0.23
sub-0055	31.7	0.25	2.9	-1.67	4.0	-1.28	5.5	0.60	3.5	-0.07	10.7	0.22	67.1	-0.21	19.8	-1.94	48.3	1.34	27	-1.12	4.0	-0.96
sub-0056	34.7	0.74	14.9	0.40	16.3	0.65	4.9	0.28	0.0	-1.58	9.9	-0.42	70.7	1.02	15.9	-2.73	16.7	-1.55	30	-0.75	3.5	-1.69
sub-0057	30.9	0.12	7.7	-0.84	0.0	-1.91	3.2	-0.64	0.5	-1.37	9.6	-0.66	55.0	-4.35	25.6	-0.77	23.2	-0.95	27	-1.12	4.3	-0.60
sub-0058	16.2	-2.31	10.9	-0.29	11.9	-0.04	4.7	0.17	5.4	0.75	9.9	-0.42	60.7	-2.40	22.7	-1.35	30.7	-0.27	37	0.11	4.5	-0.23
sub-0059	27.1	-0.51	5.6	-1.20	6.8	-0.84	6.7	1.25	6.7	1.31	10.8	0.30	68.7	0.34	23.4	-1.21	49.9	1.48	45	1.09	5.0	0.49
sub-0060	30.6	0.07	12.0	-0.10	11.7	-0.07	5.5	0.60	5.2	0.66	9.9	-0.42	70.0	0.78	35.1	1.16	41.6	0.73	44	0.97	4.0	-0.96
sub-0061	33.6	0.56	9.0	-0.61	10.7	-0.23	6.6	1.20	6.2	1.09	11.9	1.18	65.0	-0.93	33.1	0.75	27.6	-0.55	45	1.09	6.0	1.95
sub-0062	28.0	-0.36	15.6	0.52	13.7	0.24	3.4	-0.53	0.0	-1.58	11.8	1.10	71.3	1.22	34.3	0.99	39.7	0.55	38	0.23	4.5	-0.23
sub-0063	33.0	0.46	17.7	0.88	18.9	1.06	6.0	0.87	5.9	0.96	9.6	-0.66	67.7	-0.01	33.1	0.75	33.3	-0.03	38	0.23	3.8	-1.32
sub-0064	30.1	-0.01	13.6	0.17	15.8	0.57	5.5	0.60	6.1	1.05	8.5	-1.53	67.5	-0.08	29.0	-0.08	35.4	0.16	35	-0.14	4.8	0.13
sub-0065	31.1	0.15	10.0	-0.44	10.8	-0.22	6.8	1.31	3.9	0.10	10.3	-0.10	70.1	0.81	32.2	0.57	27.4	-0.57	33	-0.38	5.5	1.22
Group avg	30.2		12.6		12.2		4.4		3.7		10.4		67.7		29.4		33.6		36.1		4.7	
Stdev	6.1		5.8		6.4		1.8		2.3		1.3		2.9		4.9		11.0		8.2		0.7	

HE = healthy elder; aMCI = amnestic single domain, aMCIm = amnestic multiple domain, naMCI = non-amnestic single domain, naMCIm = non-amnestic multiple domain mild cognitive impairment; MMSE = mini-mental state examination; AVLT = Rey auditory verbal learning test; DS = Forward digit span; AMT = Attentional matrices test; TMT = Trail making test; ROCF = Rey-Osterrieth complex figure; BSRT = Babcock story recall test; FDT = Freehand drawing test; PCT = Planned copy test; RPM = Raven progressive matrices; PFT = Phonemic fluency test; SFT = Semantic fluency test; CBTT = Corsi block-tapping test; Imm = immediate; Del = delayed; * denotes that sign of Z-scores for this test has been flipped, so as positive values represent better performance (for consistency with other tests)

Supplemental Table 2: Predictive power of biochemical markers on cognitive profiles

		PC_1		PC_2		PC_3		PC_4		PC_5		PC_6		PC_7		PC_8		PC_9	
		β	p	β	p	β	p	β	p	β	p	β	p	β	p	β	p	β	p
	tHcy	.011	.9322	.312	.0199	.082	.5487	.111	.4192	-.088	.5191	-.296	.0293	-.068	.6180	-.281	.0371	.228	.0925
	Folate	-.080	.5563	.088	.5215	-.026	.8420	.013	.9234	.103	.4495	-.301	.0242	.021	.8841	.100	.4678	.123	.3677
	B_{12}	.222	.1034	-.064	.6390	.053	.7040	.078	.5676	-.023	.8654	.075	.5795	.171	.2071	.015	.9119	-.079	.5616
	B_{12} * Folate	.189	.1629	.156	.2513	-.050	.7140	.029	.8331	-.223	.0996	.078	.5718	-.117	.3926	-.038	.7781	-.024	.8622
	B_{12} * tHcy	.037	.7844	-.130	.3354	-.024	.8590	.097	.4747	-.116	.3934	-.047	.7305	.066	.6272	-.036	.7900	.179	.1889
	tHcy * Folate	.149	.2689	.173	.2082	-.020	.8788	-.105	.4390	.112	.4170	.134	.3274	.007	.9604	-.071	.6027	.018	.8876
Nuisance variables	Age	-.154	.2596	.321	.0162	-.065	.6344	-.053	.7025	.241	.0742	.245	.0697	**.431**	**.0009**	.119	.3797	-.121	.3740
	Education	.101	.4625	.120	.3810	.007	.9632	.333	.0125	-.206	.1297	.200	.1441	.346	.0097	-.255	.0586	-.145	.2828
	MMSE Scores	**.553**	**.0000**	.092	.4999	.072	.6025	.031	.8248	.208	.1285	-.140	.3042	-.042	.7674	.118	.3829	-.084	.5335
	fMRI Task Accuracy	.037	.7912	-.011	.9396	-.293	.0290	.020	.8865	.075	.5848	.340	.0114	-.029	.8342	.144	.2909	.090	.5153

		PC_{10}		PC_{11}		PC_{12}		PC_{13}		PC_{14}		PC_{15}		PC_{16}		PC_{17}	
		β	p	β	p	β	p	β	p	β	p	β	p	β	p	β	p
	tHcy	-.003	.9865	.269	.0452	-.138	.3098	.062	.6478	-.032	.8183	-.076	.5790	.046	.7356	-.074	.5872
	Folate	-.072	.5948	-.114	.4073	**-.409**	**.0021**	.044	.7490	.046	.7325	.154	.2616	.064	.6403	-.259	.0536
	B_{12}	-.165	.2252	.244	.0707	-.147	.2746	.092	.4984	.065	.6317	.007	.9567	-.014	.9225	-.005	.9769
	B_{12} * Folate	-.016	.9106	-.147	.2837	**-.480**	**.0002**	-.012	.9286	.043	.7481	.012	.9254	-.070	.6036	-.005	.9666
	B_{12} * tHcy	-.154	.2566	.089	.5164	-.139	.3076	-.058	.6689	.073	.5905	-.081	.5522	.036	.7917	.174	.1999
	tHcy * Folate	.172	.2015	-.165	.2257	-.253	.0618	.058	.6682	-.228	.0914	**.428**	**.0011**	-.031	.8190	.034	.8035
Nuisance variables	Age	.192	.1575	.096	.4859	-.175	.2004	.071	.6060	.020	.8925	.038	.7802	-.156	.2487	-.100	.4655
	Education	-.100	.4707	.040	.7681	.233	.0840	-.300	.0253	.121	.3733	.031	.8185	.013	.9224	-.096	.4799
	MMSE Scores	.084	.5347	.057	.6785	-.042	.7621	-.145	.2893	-.071	.5973	.325	.0164	.150	.2683	.247	.0674
	fMRI Task Accuracy	.014	.9126	-.019	.8964	.126	.3566	.128	.3443	-.066	.6226	-.060	.6629	-.115	.3991	-.044	.7454

Model: $PC_j = \alpha + \beta\, tHcy + \beta\, Folate + \beta\, B_{12} + \beta\, B_{12}{*}Folate + \beta\, B_{12}{*}tHcy + \beta\, tHcy{*}Folate + \beta\, Age + \beta\, Education + \beta\, MMSE + \beta\, fMRI\ task\ accuracy$; Underlined values represent $p < 0.05$ uncorrected; **Bold values** represent $p < 0.05$ Bonferroni corrected
tHcy = homocysteine; MMSE = mini-mental state examination; PC = prinicipal component

Supplemental Table 3: Predictive power of right dACC activity on cognitive profiles

		PC_1		PC_2		PC_3		PC_4		PC_5		PC_6		PC_7		PC_8		PC_9	
		β	p	β	p	β	p	β	p	β	p	β	p	β	p	β	p	β	p
	Right dACC	.188	.1467	-.091	.4885	.009	.9404	.120	.3541	-.188	.1484	.065	.6136	.210	.1049	.000	.9907	-.100	.4449
Nuisance variables	Age	-.233	.0713	.300	.0192	-.059	.6519	-.030	.8231	.267	.0384	.135	.2945	**.446**	**.0003**	.100	.4377	-.025	.8428
	Education	.104	.4276	.125	.3387	-.012	.9297	.310	.0151	-.179	.1683	.202	.1166	.356	.0047	-.211	.1034	-.166	.2030
	MMSE Scores	**.565**	**.0000**	.114	.3824	.079	.5430	.050	.7044	.148	.2554	-.136	.2913	-.055	.6728	.117	.3704	-.145	.2696
	fMRI Task Accuracy	-.001	.9918	-.022	.8649	-.300	.0197	.059	.6549	.020	.8758	.263	.0419	-.045	.7291	.130	.3137	.153	.2399

		PC_{10}		PC_{11}		PC_{12}		PC_{13}		PC_{14}		PC_{15}		PC_{16}		PC_{17}	
		β	p	β	p	β	p	β	p	β	p	β	p	β	p	β	p
	Right dACC	-.048	.7118	**.388**	**.0023**	-.082	.5253	-.051	.6965	-.071	.5929	.025	.8487	-.059	.6534	.052	.6842
Nuisance variables	Age	.167	.1986	.142	.2737	-.123	.3442	.057	.6638	.027	.8299	.008	.9541	-.116	.3723	-.094	.4723
	Education	-.076	.5603	-.023	.8642	.152	.2405	-.302	.0186	.095	.4629	.117	.3649	.007	.9600	-.137	.2872
	MMSE Scores	.092	.4854	.113	.3870	-.019	.8836	-.154	.2365	-.058	.6575	.259	.0431	.127	.3287	.211	.1056
	fMRI Task Accuracy	-.027	.8437	.007	.9592	.098	.4504	.099	.4540	-.025	.8533	-.140	.2820	-.099	.4462	-.036	.7876

Model: $PC_j = \alpha + \beta$ Right dACC + β Age + β Education + β MMSE + β fMRI task accuracy; Underlined values represent $p < 0.05$ uncorrected; **Bold values** represent $p < 0.05$ Bonferroni corrected

dACC = dorsal anterior cingulate cortex; MMSE = mini-mental state examination; PC = prinicipal component

Supplemental Table 4: Predictive power of right vmPFC activity on cognitive profiles

		PC_1		PC_2		PC_3		PC_4		PC_5		PC_6		PC_7		PC_8	
		β	p	β	p	β	p	β	p	β	p	β	p	β	p	β	p
	Right vmPFC	-.007	.9604	-.025	.8451	-.052	.6892	.022	.8620	.031	.8062	.062	.6341	.149	.2508	.040	.7611
Nuisance variables	Age	-.232	.0702	.304	.0169	-.050	.7041	-.034	.7977	.261	.0423	.125	.3374	**.420**	**.0009**	.093	.4754
	Education	.088	.4979	.135	.2998	-.009	.9451	.299	.0200	-.165	.2020	.192	.1394	.328	.0095	-.214	.0998
	MMSE Scores	**.535**	**.0000**	.128	.3270	.077	.5514	.031	.8160	.179	.1698	-.146	.2661	-.086	.5121	.117	.3671
	fMRI Task Accuracy	-.009	.9473	-.022	.8670	-.308	.0165	.057	.6628	.031	.8165	.269	.0362	-.032	.8043	.136	.2976

		PC_9		PC_{10}		PC_{11}		PC_{12}		PC_{13}		PC_{14}		PC_{15}		PC_{16}		PC_{17}	
		β	p	β	p	β	p	β	p	β	p	β	p	β	p	β	p	β	p
	Right vmPFC	.096	.4595	.009	.9406	.103	.4298	**.380**	**.0028**	.053	.6816	.038	.7747	-.077	.5516	-.122	.3448	-.037	.7742
Nuisance variables	Age	-.041	.7506	.166	.2021	.124	.3362	-.189	.1425	.048	.7169	.021	.8691	.022	.8624	-.095	.4639	-.088	.5021
	Education	-.164	.2060	-.073	.5772	-.064	.6220	.132	.3106	-.301	.0187	.099	.4490	.121	.3499	.021	.8719	-.138	.2878
	MMSE Scores	-.127	.3303	.100	.4448	.053	.6830	.001	.9933	-.145	.2654	-.047	.7239	.254	.0484	.135	.3015	.202	.1204
	fMRI Task Accuracy	.170	.1888	-.024	.8547	.007	.9600	.151	.2407	.108	.4091	-.017	.8988	-.151	.2447	-.114	.3856	-.043	.7453

Model: $PC_j = \alpha + \beta$ Right vmPFC + β Age + β Education + β MMSE + β fMRI task accuracy; Underlined values represent $p < 0.05$ uncorrected; **Bold values** represent $p < 0.05$ Bonferroni corrected

Supplemental Table 4: Predictive power of bilateral vmPFC activity on cognitive profiles

		PC_1		PC_2		PC_3		PC_4		PC_5		PC_6		PC_7		PC_8	
		β	p	β	p	β	p	β	p	β	p	β	p	β	p	β	p
	Bilateral vmPFC	-.058	.6585	-.093	.4777	-.047	.7171	.057	.6620	.031	.8171	-.037	.7756	.009	.9498	.074	.5690
Nuisance variables	Age	-.220	.0885	.321	.0110	-.048	.7178	-.043	.7471	.260	.0425	.144	.2699	**.444**	**.0005**	.083	.5209
	Education	.095	.4723	.145	.2639	-.007	.9600	.293	.0233	-.167	.1987	.202	.1193	.337	.0081	-.220	.0859
	MMSE Scores	**.533**	**.0000**	.125	.3307	.076	.5682	.033	.8026	.179	.1697	-.148	.2558	-.088	.4949	.120	.3594
	fMRI Task Accuracy	-.011	.9300	-.024	.8560	-.303	.0178	.057	.6597	.028	.8289	.259	.0422	-.052	.6904	.134	.2988

		PC_9		PC_{10}		PC_{11}		PC_{12}		PC_{13}		PC_{14}		PC_{15}		PC_{16}		PC_{17}	
		β	p	β	p	β	p	β	p	β	p	β	p	β	p	β	p	β	p
	Bilateral vmPFC	-.024	.8591	.017	.8903	.096	.4650	.295	.0215	-.071	.5812	.070	.5971	-.140	.2855	-.083	.5246	-.160	.2153
Nuisance variables	Age	-.019	.8874	.163	.2063	.119	.3572	-.192	.1382	.073	.5761	.011	.9305	.041	.7560	-.097	.4599	-.057	.6658
	Education	-.154	.2344	-.074	.5679	-.069	.5981	.121	.3528	-.288	.0250	.092	.4804	.133	.3039	.023	.8608	-.120	.3566
	MMSE Scores	-.130	.3167	.100	.4440	.055	.6725	.006	.9677	-.149	.2578	-.044	.7398	.250	.0535	.133	.3034	.197	.1276
	fMRI Task Accuracy	.156	.2340	-.024	.8540	-.002	.9847	.116	.3724	.097	.4600	-.018	.8902	-.148	.2549	-.102	.4362	-.046	.7274

Model: $PC_j = \alpha + \beta$ Bilateral vmPFC + β Age + β Education + β MMSE + β fMRI task accuracy; Underlined values represent $p < 0.05$ uncorrected; **Bold values** represent $p < 0.05$ Bonferroni corrected

vmPFC = ventromedial prefrontal cortex; MMSE = mini-mental state examination; PC = prinicipal component

Supplemental Table 5: Predictive power of right dACC activity on cognitive profiles, controlling for vitamin B_{12}

		PC_1		PC_2		PC_3		PC_4		PC_5		PC_6		PC_7		PC_8	
		β	p	β	p	β	p	β	p	β	p	β	p	β	p	β	p
	Right dACC	.101	.4393	-.017	.8942	-.007	.9574	.109	.4055	-.238	.0672	.014	.9118	.147	.2644	-.055	.6795
	B_{12}	.169	.1970	-.143	.2771	.031	.8136	.021	.8742	.097	.4600	.099	.4610	.123	.3512	.105	.4241
Nuisance variables	Age	-.210	.1081	.280	.0298	-.055	.6761	-.027	.8346	.280	.0322	.149	.2564	**.463**	**.0001**	.114	.3860
	Education	.108	.4107	.121	.3526	-.011	.9339	.311	.0157	-.176	.1793	.205	.1183	.359	.0049	-.208	.1104
	MMSE Scores	**.559**	**.0000**	.119	.3630	.078	.5571	.049	.7108	.145	.2707	-.140	.2830	-.060	.6491	.113	.3915
	fMRI Task Accuracy	.021	.8685	-.041	.7543	-.296	.0213	.061	.6353	.033	.8094	.276	.0331	-.028	.8283	.144	.2755

		PC_9		PC_{10}		PC_{11}		PC_{12}		PC_{13}		PC_{14}		PC_{15}		PC_{16}		PC_{17}	
		β	p	β	p	β	p	β	p	β	p	β	p	β	p	β	p	β	p
	Right dACC	-.078	.5595	.080	.5450	**.392**	**.0022**	.015	.9065	-.118	.3729	-.164	.2102	.027	.8416	-.072	.5835	.052	.6881
	B_{12}	-.043	.7461	-.247	.0579	-.008	.9550	-.188	.1510	.130	.3224	.182	.1643	-.004	.9746	.025	.8494	.000	.9932
Nuisance variables	Age	-.031	.8168	.134	.3085	.141	.2822	-.149	.2595	.074	.5732	.052	.6959	.008	.9535	-.113	.3956	-.095	.4716
	Education	-.167	.2032	-.083	.5247	-.023	.8665	.147	.2598	-.298	.0199	.100	.4443	.117	.3771	.008	.9538	-.137	.2968
	MMSE Scores	-.143	.2745	.101	.4437	.114	.3871	-.012	.9324	-.159	.2269	-.065	.6242	.259	.0465	.126	.3376	.211	.1040
	fMRI Task Accuracy	.148	.2574	-.059	.6508	.006	.9649	.073	.5831	.116	.3793	-.001	.9948	-.141	.2821	-.096	.4667	-.036	.7865

Model: $PC_j = \alpha + \beta$ Right dACC + $\beta B_{12} + \beta$ Age + β Education + β MMSE + β fMRI task accuracy; Underlined values represent $p < 0.05$ uncorrected; **Bold values** represent $p < 0.05$ Bonferroni corrected
dACC = dorsal anterior cingulate cortex; MMSE = mini-mental state examination; PC = prinicipal component

Supplemental Table 6: Predictive power of right vmPFC activity on cognitive profiles, controlling for B_{12} * Folate interaction

		PC_1		PC_2		PC_3		PC_4		PC_5		PC_6		PC_7		PC_8	
		β	p	β	p	β	p	β	p	β	p	β	p	β	p	β	p
	Right vmPFC	.085	.5201	.074	.5741	-.087	.5077	.041	.7529	-.094	.4762	.130	.3208	.104	.4293	.037	.7768
	B_{12} * Folate	.191	.1418	.208	.1108	-.073	.5738	.040	.7599	-.260	.0470	.142	.2769	-.095	.4695	-.006	.9622
Nuisance variables	Age	-.202	.1210	.337	.0092	-.061	.6428	-.028	.8348	.221	.0900	.147	.2663	**.405**	**.0015**	.092	.4752
	Education	.076	.5740	.121	.3529	-.004	.9786	.296	.0208	-.148	.2572	.183	.1602	.334	.0089	-.213	.1025
	MMSE Scores	**.510**	**.0001**	.101	.4421	.086	.5088	.026	.8471	.213	.1017	-.164	.2090	-.074	.5766	.118	.3705
	fMRI Task Accuracy	-.006	.9577	-.020	.8773	-.309	.0171	.058	.6597	.027	.8329	.271	.0359	-.033	.8025	.136	.3009

		PC_9		PC_{10}		PC_{11}		PC_{12}		PC_{13}		PC_{14}		PC_{15}		PC_{16}		PC_{17}	
		β	p	β	p	β	p	β	p	β	p	β	p	β	p	β	p	β	p
	Right vmPFC	.062	.6338	.017	.9039	.060	.6461	.284	.0298	.069	.6090	.090	.4981	-.146	.2634	-.211	.1050	-.091	.4906
	B_{12} * Folate	-.072	.5897	.016	.9006	-.090	.4941	-.201	.1231	.032	.8106	.110	.4012	-.144	.2738	-.185	.1594	-.112	.3931
Nuisance variables	Age	-.053	.6898	.168	.2000	.110	.4052	-.221	.0917	.053	.6866	.038	.7727	-.001	.9965	-.124	.3457	-.106	.4182
	Education	-.160	.2223	-.074	.5739	-.058	.6566	.145	.2700	-.303	.0189	.092	.4832	.130	.3210	.033	.8060	-.131	.3172
	MMSE Scores	-.118	.3725	.098	.4593	.065	.6212	.027	.8366	-.149	.2518	-.061	.6464	.273	.0347	.159	.2240	.217	.0950
	fMRI Task Accuracy	.169	.2012	-.024	.8634	.006	.9616	.149	.2581	.108	.4078	-.016	.9081	-.153	.2435	-.116	.3756	-.044	.7373

Model: $PC_j = \alpha + \beta$ Right vmPFC + β B_{12}*Folate + β Age + β Education + β MMSE + β fMRI task accuracy; Underlined values represent $p < 0.05$ uncorrected; **Bold values** represent $p < 0.05$ Bonferroni corrected

Supplemental Table 6: Predictive power of bilateral vmPFC activity on cognitive profiles, controlling for tHcy * Folate interaction

		PC_1		PC_2		PC_3		PC_4		PC_5		PC_6		PC_7		PC_8	
		β	p	β	p	β	p	β	p	β	p	β	p	β	p	β	p
	Bilateral vmPFC	-.010	.9373	-.086	.5107	-.069	.6006	.012	.9267	.107	.4130	.053	.6914	.037	.7781	.066	.6137
	tHcy * Folate	.114	.3817	.016	.9079	-.052	.6973	-.106	.4170	.183	.1629	.213	.1012	.067	.6090	-.017	.8917
Nuisance variables	Age	-.227	.0833	.320	.0119	-.045	.7345	-.037	.7815	.248	.0574	.131	.3210	**.439**	**.0003**	.084	.5244
	Education	.078	.5578	.143	.2814	.001	.9920	.309	.0168	-.195	.1359	.169	.1990	.327	.0108	-.218	.0941
	MMSE Scores	**.556**	**.0000**	.128	.3293	.065	.6221	.011	.9396	.217	.0938	-.104	.4270	-.074	.5741	.116	.3793
	fMRI Task Accuracy	.010	.9345	-.021	.8823	-.313	.0153	.038	.7744	.062	.6377	.298	.0210	-.039	.7711	.131	.3193

		PC_9		PC_{10}		PC_{11}		PC_{12}		PC_{13}		PC_{14}		PC_{15}		PC_{16}		PC_{17}	
		β	p	β	p	β	p	β	p	β	p	β	p	β	p	β	p	β	p
	Bilateral vmPFC	-.022	.8628	.102	.4453	.046	.7290	.316	.0143	-.071	.5898	-.031	.8186	.032	.8106	-.113	.3920	-.133	.3110
	tHcy * Folate	.003	.9787	.202	.1245	-.119	.3737	.049	.7110	.001	.9875	-.240	.0653	**.409**	**.0015**	-.070	.5949	.065	.6223
Nuisance variables	Age	-.019	.8803	.151	.2482	.127	.3378	-.195	.1344	.073	.5804	.026	.8477	.016	.9129	-.093	.4826	-.061	.6435
	Education	-.155	.2342	-.106	.4189	-.050	.7034	.113	.3899	-.288	.0254	.130	.3241	.070	.5944	.034	.7990	-.130	.3180
	MMSE Scores	-.129	.3257	.142	.2786	.031	.8175	.016	.9014	-.148	.2559	-.094	.4735	.334	.0098	.119	.3636	.210	.1078
	fMRI Task Accuracy	.156	.2335	.013	.9227	-.024	.8610	.125	.3432	.097	.4626	-.062	.6379	-.074	.5790	-.114	.3815	-.034	.7993

Model: $PC_j = \alpha + \beta$ Bilateral vmPFC + β tHcy*Folate + β Age + β Education + β MMSE + β fMRI task accuracy; Underlined values represent $p < 0.05$ uncorrected; **Bold values** represent $p < 0.05$ Bonferroni corrected

tHcy = homocysteine; vmPFC = ventromedial prefrontal cortex; MMSE = mini-mental state examination; PC = prinicipal component

Supplemental Table 7: Predictive power of fMRI task accuracy on cognitive profiles

	PC_1		PC_2		PC_3		PC_4		PC_5		PC_6		PC_7		PC_8		PC_9	
	β	p	β	p	β	p	β	p	β	p	β	p	β	p	β	p	β	p
fMRI Task Accuracy	-.008	.9547	-.019	.8794	-.301	.0177	.054	.6735	.027	.8410	.261	.0406	-.052	.6860	.130	.3116	.157	.2243
Age	-.233	.0677	.300	.0189	-.059	.6485	-.030	.8164	.267	.0367	.135	.2958	**.446**	**.0003**	.100	.4355	-.025	.8508
Education	.088	.5040	.133	.3080	-.013	.9190	.300	.0177	-.163	.2057	.197	.1261	.338	.0067	-.211	.0993	-.157	.2234
MMSE Scores	**.535**	**.0000**	.129	.3178	.077	.5486	.031	.8073	.178	.1660	-.147	.2587	-.089	.4880	.117	.3681	-.129	.3175

	PC_{10}		PC_{11}		PC_{12}		PC_{13}		PC_{14}		PC_{15}		PC_{16}		PC_{17}	
	β	p	β	p	β	p	β	p	β	p	β	p	β	p	β	p
fMRI Task Accuracy	-.025	.8543	-.007	.9601	.101	.4394	.101	.4308	-.022	.8639	-.141	.2766	-.097	.4540	-.038	.7710
Age	.167	.1998	.142	.2754	-.123	.3366	.057	.6591	.027	.8332	.008	.9506	-.116	.3695	-.095	.4650
Education	-.072	.5768	-.056	.6627	.159	.2164	-.297	.0190	.102	.4295	.115	.3739	.012	.9262	-.141	.2752
MMSE Scores	.100	.4389	.051	.6886	-.006	.9662	-.146	.2585	-.047	.7201	.255	.0446	.137	.2931	.203	.1165

Model: $PC_j = \alpha + \beta$ fMRI task accuracy + β Age + β Education + β MMSE; Underlined values represent $p < 0.05$ uncorrected; **Bold values** represent $p < 0.05$ Bonferroni corrected

MMSE = mini-mental state examination; PC = prinicipal component
