# Supplementary material for: Brain Hemodynamic Intermediate Phenotype Links Vitamin B12 to Cognitive Profile of Healthy and Mild Cognitive Impaired Subjects
Source: Neural Plast. 2019 Jun 2;2019:6874805. doi: 10.1155/2019/6874805 (PMC6589271; doi:10.1155/2019/6874805)
Supplement: Supplementary 2 — Complete list of the members and affiliations of the Train the Brain Consortium. [file 6874805.f2.pdf]

Supplementary 2

Complete list of the members and affiliations of the Train the Brain Consortium

L. Maffei[1], E. Picano[2], M.G. Andreassi[2], A. Angelucci[1], F. Baldacci[3], L. Baroncelli[1], T. Begenisic[1], P.F. Bellinvia[2], N. Berardi[1], L. Biagi[4], J. Bonaccorsi[1], E. Bonanni[3], U. Bonuccelli[3], A. Borghini[2], C. Braschi[1], M. Broccardi[1], R.M. Bruno[2,6], M. Caleo[1], C. Carlesi[1,3], L. Carnicelli[3], G. Cartoni[3], M.C. Cenni[1], R. Ceravolo[3], L. Chico[3], S. Cintoli[1,3], G. Cioni[4,6], M. Coscia[1], M. Costa[1], G. D'Angelo[3], P. D'Ascanio[7], M. De Nes[2], S. Del Turco[2], E. Di Coscio[7], M. Di Galante[7], N. di Lascio[2], F. Faita[2], I. Falorni[1,3], U. Faraguna[4,7], A. Fenu[2], L. Fortunato[2], R. Franco[1], L. Gargani[2], R. Gargiulo[1], L. Ghiadoni[3], F.S. Giorgi[3], R. Iannarella[3], C. Iofrida[5], C. Kusmic[2], F. Limongi[1], M. Maestri[3], M. Maffei[2,6], S. Maggi[1], M. Mainardi[1], L. Mammana[1], A. Marabotti[3], V. Mariotti[7], E. Melissari[5], A. Mercuri[2], S. Micera[8,9], S. Molinaro[2], R. Narducci[1], T. Navarra[2], M. Noale[1], C. Pagni[3], S. Palumbo[5], R. Pasquariello[4], T. Pizzorusso[1], A. Poli[1], L. Pratali[2], A. Retico[10], G. Rota[5], A. Sale[1], S. Sbrana[2], G. Scabia[6], M. Scali[1], D. Scelfo[4], R. Sicari[2], G. Siciliano[3], F. Stea[2], S. Taddei[6], G. Tognoni[3], A. Tonacci[2], M. Tosetti[4], S. Turchi[2], L. Volpi.[1,3]

[1] Neuroscience Institute, CNR, Via G. Moruzzi 1, 56124 Pisa, Italy.
[2] Institute of Clinical Physiology of the CNR, Via G. Moruzzi 1, 56100 Pisa, Italy.
[3] Department of Clinical and Experimental Medicine-Neurology Unit, University of Pisa & AOU Pisa, Italy.
[4] IRCCS Stella Maris, Viale del Tirreno 341, Calambrone, Italy.
[5] Department of Surgical, Medical, Molecular Pathology and of Critical Care, University of Pisa, Via Savi 10, 56126 Pisa, Italy.
[6] Department of Clinical and Experimental Medicine, University of Pisa, Via Savi 10, 56126 Pisa, Italy.
[7] Department of Translational research and New technologies in Medicine and Surgery, University of Pisa, Via Savi 10, 56126 Pisa, Italy.
[8] Bertarelli Foundation Chair in Translational Neuroengineering, Center for Neuroprosthetics and Institute of Bioengineering, Ecole Polytechnique Federale de Lausanne, CH-1015 Lausanne, Switzerland.
[9] Scuola Superiore Sant'Anna, P.za Martiri della Libertà 33, 56127 Pisa, Italy.
[10] National Institute of Nuclear Physics (INFN), Pisa section, Largo B. Pontecorvo, 3, 56127 Pisa, Italy.
